# Unbiased pattern analysis reveals highly diverse responses of cytoskeletal systems to cyclic straining

**DOI:** 10.1371/journal.pone.0210570

**Published:** 2019-03-13

**Authors:** Ronald Springer, Alexander Zielinski, Catharina Pleschka, Bernd Hoffmann, Rudolf Merkel

**Affiliations:** Institute of Complex Systems 7, Forschungszentrum Jülich GmbH, Jülich, Germany; Institut de Genetique et Developpement de Rennes, FRANCE

## Abstract

In mammalian cells, actin, microtubules, and various types of cytoplasmic intermediate filaments respond to external stretching. Here, we investigated the underlying processes in endothelial cells plated on soft substrates from silicone elastomer. After cyclic stretch (0.13 Hz, 14% strain amplitude) for periods ranging from 5 min to 8 h, cells were fixed and double-stained for microtubules and either actin or vimentin. Cell images were analyzed by a two-step routine. In the first step, micrographs were segmented for potential fibrous structures. In the second step, the resulting binary masks were auto- or cross-correlated. Autocorrelation of segmented images provided a sensitive and objective measure of orientational and translational order of the different cytoskeletal systems. Aligning of correlograms from individual cells removed the influence of only partial alignment between cells and enabled determination of intrinsic cytoskeletal order. We found that cyclic stretching affected the actin cytoskeleton most, microtubules less, and vimentin mostly only via reorientation of the whole cell. Pharmacological disruption of microtubules had barely any influence on actin ordering. The similarity, i.e., cross-correlation, between vimentin and microtubules was much higher than the one between actin and microtubules. Moreover, prolonged cyclic stretching slightly decoupled the cytoskeletal systems as it reduced the cross-correlations in both cases. Finally, actin and microtubules were more correlated at peripheral regions of cells whereas vimentin and microtubules correlated more in central regions.

## Introduction

Within the organism most tissue cells are permanently exposed to mechanical deformation. For example, cells of the myocard experience strains of up to 30% with each heart beat [1] and cells lining the alveoli of the lung experience similar strains during breathing [2]. Even larger strains, of up to 80%, have been inferred for soft tissues of the shoulder as a result of carrying a backpack [3]. Consequently, most tissues exhibit structures that are clearly adapted to these intense mechanical deformations. Obviously, cells embedded in these tissues must sense the mechanical signal and adapt to it. In cases where these cellular adaptations to mechanical strain are compromised or maladapted, severe pathological disorders like enlargement of cerebral aneurysms [4] and right heart failure in response to pulmonary arterial hypertension [5] occur. Thus, the interplay of tissue cells and mechanical signals is of high interest.

Unraveling the processes underlying cellular reactions to deformation is a challenging task, as it is very difficult to apply well-defined mechanical signals and to quantify the ensuing responses. This challenge can be met in experiments on cells cultivated on elastomeric substrates undergoing uniaxial or biaxial strain [6–10] because here substrate strain can be carefully controlled and cellular reactions can be well studied by most techniques of molecular cell biology.

Cell reactions to applied stretch have recently been reviewed [11]. The most obvious response to cyclic substrate strain is reorientation of the cell body and of the cytoskeletal systems endowing the cell with mechanical stiffness and with the ability to adhere to and tense its substrate. This process is most likely driven by the need to re-establish mechanical homeostasis and depends on substrate stiffness, strain amplitude, strain frequency, and the exact waveform of the applied repetitive strain [8, 10, 12, 13]. While all three cytoskeletal systems (microtubules, cytoplasmic intermediate filaments and actin) clearly undergo reorientation [14], the distinct processes differ greatly. At present, much evidence points towards a dominant role of actin stress fibers. For example, Dartsch et al. [7] commented on the massive changes of actin cytoskeletal structure while microtubules just rotate. Wang et al. [15] reported that contractility of stress fibers is mandatory for cell reorientation. Moreover, Iba et al. [14] and Goldyn et al. [16] described that pharmacological weakening of the actin cytoskeleton abrogates cellular reorientation whereas depolymerization of microtubules has no effect on reorientation itself but accelerates reorientation [17]. Beyond these generalized effects, local orientations of actin fibers and microtubules have been determined revealing that cyclic stretch increases the correlation of these fiber directions. These findings have led to the hypothesis of strain dependent functional association between those two cytoskeletal networks [17].

Cytoskeletal reorientation has been mostly studied via measurement of the direction of the fibers forming the respective cytoskeleton. Yet, fluorescence micrographs clearly point to an additional strain-induced increase of order, especially in the actin cytoskeleton. Unfortunately, quantification of cytoskeletal order is a demanding task. Several studies that tackled this problem adopted a two-step approach. First, they traced filaments in immunfluorescence micrographs and, subsequently, they calculated parameters characterizing cytoskeletal structure and order. While there are surprisingly many methods to extract centerlines of fibrous structures from images, cf. Eltzner et al., [18] and references therein, most authors have used algorithms based on correlating the fluorescent image suffering from noise and limited resolution with a synthetic image of a short fiber. In these methods, the correlation coefficient is used as likelihood for positions and orientations of fibers. This approach was elegantly pioneered by Lichtenstein et al. [19] and later substantially expanded by Shah [20] and Basu [21]. Examples for parameters used to characterize cytoskeletal networks are fiber length and distance between branches [20, 22], the order parameter known from liquid crystal physics [23–25], and a generalization of the latter capable of capturing the orientational correlation between two sets of filaments [26]. As an alternative to network extraction, methods from texture analysis have also been widely used. Here, fibrous structures are identified by edge-sensitive filters [27], Fourier transform methods [16, 28], or the characteristic local variation of gray values, i.e., by gray value gradients and the structure tensor derived from those [8, 29, 30].

Exploiting such methods it was found that cytoskeletal order is clearly dependent on mechanical signals. For example, during wound healing stress fiber orientation is clearly non-random with respect to the long axis of the wound [28]. In addition, actin cytoskeletal order depends on the mechanical treatment of the wound [31]. Moreover, substrate stiffness determines the liquid crystalline order parameter of the stress fiber network in mesenchymal stem cells [24, 25] and in a cell line a sudden alteration of substrate stiffness was shown to induce a delayed but pronounced decay of the same parameter [23]. Even closer related to our work, some authors report on the order of the actin cytoskeleton in cells undergoing cyclic strain. Cardiac fibroblasts respond to equiaxial, cyclic strain by changes of the fractal dimension of their actin cytoskeleton [32] and a fibroblast line was shown to increase the parallel alignment of their stress fibers upon cyclic uniaxial strain [33].

Taken together, there is compelling evidence that cytoskeletal structure and order change in response to mechanical signals. However, a systematic and quantitative study of the relation between the different cytoskeletal systems is still missing. Here we set out to explore the influence of cyclic straining on all three cytoskeletal systems. To do so we cultivated human umbilical cord venous endothelial cells (HUVECs) on silicone lamellae and exposed them to cyclic strain of 14% amplitude and 130 mHz frequency. Cells were fixed, stained for microtubules and simultaneously for either actin or vimentin, and imaged by light microscopy. Because the structures of the three cytoskeletal systems are extremely different with respect to connectivity, straightness, inter-filament distances and crossings, none of the above mentioned methods to quantify cytoskeletal structure seemed capable of dealing with all cytoskeletal systems without massive modifications for each. Therefore we saw the need for a generic, conceptually simple method and resorted to auto- and cross-correlation analysis, a mathematical method from statistics widely used in physics to detect and quantify order in noisy and seemingly random data [34, 35].

With this tool we tackled the following questions: As individual cells exhibit cytoskeletal order even before straining, does cyclic substrate straining simply align the individual cells or does it also increase the order in each cell? Is strain-induced ordering different for the three cytoskeletal systems? And, finally, how large are the influences of the individual cytoskeletal systems on each other?

The same fluorescence micrographs analyzed here for cytoskeletal order have been already used to explore reorientation, i.e., rotation, of the cytoskeletal systems with established algorithms [36]. Therefore this aspect will be largely ignored here.

## Materials and methods

### Cell straining

Cell straining was performed in chambers from silicone elastomer (Sylgard 184; Dow Corning, Auburn MI; Base to cross-linker ratio 40:1 with a Young’s modulus of 50 kPa). Chamber manufacturing was done as described before [8] with the only exception that the microstructure molded into the chamber bottom was reduced to two narrow lines of microdots at the outer edges of the analysis region (inner square centimeter of a 2 cm wide rectangular chamber). This still enabled exact alignment of samples during microscopy but excluded contact guidance. Chambers were coated with human fibronectin (BD biosciences) by 30 min incubation at 37 °C with a 20 μg/mL solution in phosphate buffered saline (PBS, 137 mM NaCl, 2.7 mM KCl, 1.47 mM KH_2_PO_4_, 8.1 mM Na_2_HPO_4_, pH 7.4) and subsequent washing with the same buffer.

### Cell culture

Primary HUVEC cells (order number C2519A from Lonza) were cultivated at 37 °C and 5% CO_2_ in humidified atmosphere. Cultivation took place in endothelial growth medium 2 (EGM-2; produced from Endothelial Basal Medium 2 and the SingleQuots Supplement; both Lonza). Only cells of passages 1 to 5 were used. In a fibronectin coated straining chamber 15000 cells were seeded. Following an adhesion period of 16 h, cell straining (14% amplitude, 130 mHz frequency, trapezoidal approximation of a sine wave) was applied for predefined durations.

In some experiments microtubules were destabilized by 10 μM nocodazole (Sigma). It was added from a 1 mM stock solution in DMSO (dimethyl sulfoxide) 10 min before stretch and remained present throughout. Controls for these data sets were cells treated with DMSO alone.

### Immunocytochemistry

Immunocytochemical labeling was done immediately after straining. For actin and microtubule staining cells were fixed for 30 min at 37 °C with 3.7% paraformaldehyde (Merck) in cytoskeleton buffer (CB; 150 mM NaCl, 5 mM MgCl_2_, 5 mM glucose, 5 mM EGTA, 10 mM MES; all from Sigma; pH 6.1). The reaction was quenched with glycine (Sigma; 10 min, 30 mM in CB) and cell membranes were permeabilized with 0.2% Triton-X in CB for 10 min. After three times washing with CB, samples were blocked with 10% goat serum (Sigma) in CB for 30 min. Cells used for immunostainings of microtubules and vimentin were fixed with methanol (Merck) at -20 °C for 10 min and directly blocked with 5% BSA in CB for 30 min. After rinsing all samples with CB, primary antibodies were added for 1 h at 37 °C. Unbound antibodies were removed by washing three times in CB with 0.2% Tween-20 (Sigma) for 5 min, followed by incubation with secondary antibodies in CB for 60 min at 37 °C. Primary and secondary antibodies were diluted (1:100) in either 2% goat serum or 1% BSA in CB. Samples were washed in CB and rinsed with water. Subsequently, elastomer chamber bottoms were attached to a microscope slide and chamber walls removed with a scalpel. For best imaging, samples were mounted with Fluoromount (Sigma) containing 0.1% 1,4-diazabicyclo[2.2.2]octane (Sigma) and overlaid with a cover slip. Primary antibodies used were directed against tubulin (clone YL ½ [MAB1864], Millipore) and vimentin (clone VIM-13.2 [V5255], Sigma). Corresponding secondary antibodies were coupled to Cy2 (F(ab)_2_ fragment; Dianova) or to Alexa Fluor 647 (Invitrogen). The actin cytoskeleton was stained with Alexa Fluor 546 labelled phalloidin (Invitrogen).

### Microscopy

Microscopy was done on an inverse confocal laser scanning microscope (LSM510, Carl Zeiss, Jena, Germany) equipped with an oil immersion lens (PlanNeofluoar 40x/1.30 Ph3, Zeiss). Alexa Fluor 488 was excited with the 488 nm line of an argon ion laser and observed through a band-pass filter (505 nm-530 nm), Cy3 with a 543 nm HeNe laser (band-pass 560 nm-614 nm) and Alexa Fluor 647 with a 633 nm HeNe laser (long pass 650 nm). Pixel size ranged from 101 nm to 167 nm, with the majority of micrographs acquired at a pixel size of 112 nm. For analysis the images were therefore interpolated to a common pixel size of 112 nm.

### Statistical analysis

Statistical significance was tested by the non-parametric Kolmogorov-Smirnov (KS) test [37]. We report the resulting P value, that is, the probability that the measured distributions of data points arose from the same underlying probability distribution. Moreover, the effect size was characterized by Hedges’ g [38] defined as $g=b\frac{\langle x_{1}\rangle -\langle x_{2}\rangle }{s_{p}}$ where <x_n_> denotes the mean value of the nth data set, $s_{p}=\sqrt{\frac{\left(n_{1}-1\right)s_{1}^{2}+\left(n_{2}-1\right)s_{2}^{2}}{n_{1}+n_{2}-2}}$ the standard deviation of the pooled data sets consisting of n_i_ samples with standard variations s_i_. The correction factor b to remove statistical bias is given by $b=1-\frac{3}{4\left(n_{1}+n_{2}\right)-9}$ and for our sample sizes (>51) very close to 1.

## Results

**Fig 1:**
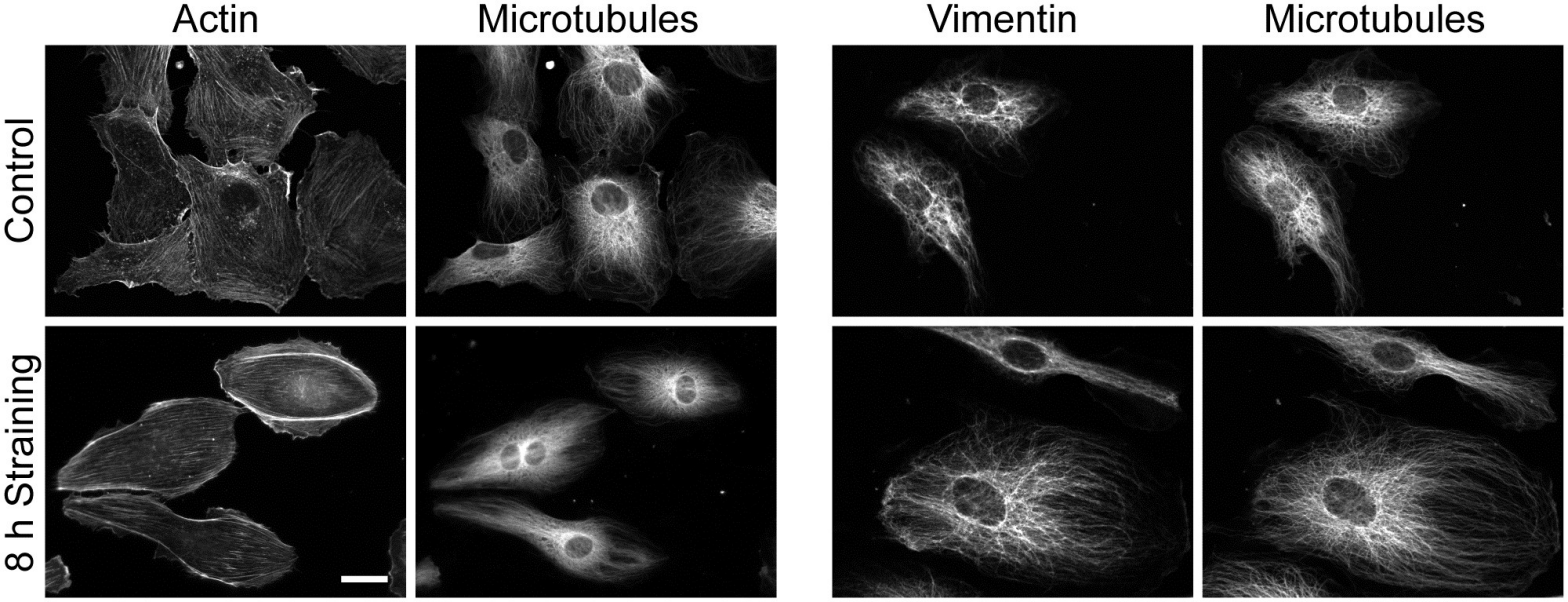
External straining aligns the cell cytoskeleton. Immunofluorescence micrographs of actin and microtubules (left) and vimentin and microtubules (right) in control cells (top) and cells strained for 8 h at 14% and 130 mHz (bottom). Stretch direction is vertical. Image pairs show identical cells. Scale bar, 20 μm, applies to all.

Human umbilical cord vein cells were strained for predefined periods and stained for either actin and microtubules or vimentin and microtubules. Micrographs, cf. Fig 1, clearly showed rotation of cytoskeletal fibers and cell outlines away from the direction of strain. Moreover, the images pointed towards increased alignment and reinforcement, especially of the actin cytoskeleton.

### Image processing and data analysis algorithms

Our first task was to find an evaluation method capable of quantifying cytoskeletal order. Especially the analysis of the finely structured microtubule and vimentin patterns (Fig 1) was challenging. As here many intersections of fibrous structures occur, all algorithms relying on edge detection (e.g. the structure tensor approach; [8, 30] or Sobel filters [27]) will give uncertain results. Moreover, in these two cytoskeletal systems it appeared impossible to trace individual filaments or fiber bundles as is established for the actin cytoskeleton [19, 21]. Therefore we resorted to correlation analysis. However, autocorrelating the fluorescence micrographs directly gave results that were very difficult to interpret. We also tried Fourier transforms of these images to determine cytoskeletal order [28] but to no better avail. Most likely reasons for these problems were inhomogeneous staining, debris and bright artifacts around the nucleus that together obscured all Fourier space or correlation patterns originating from cytoskeletal systems.

For this reason we introduced a processing step to extract cytoskeletal, i.e. fibrous structures. To this end, micrographs were segmented by a two-step algorithm. In the first step, we determined a cell mask by the triangle segmentation algorithm of Zack et al. [39]. This algorithm uses the histogram of gray values for foreground-background separation. In detail, in the histogram a line is drawn from the absolute maximum to the highest gray value occurring in the figure and the distance of each point on the histogram to this line is determined. The gray value of the histogram point with the highest distance to this line is chosen as threshold for segmentation.

In the second step, we had to separate cytoskeletal structures from artifacts. For this separation we used the fact that cytoskeletal structures are of small dimensions, at least in one direction. Thus, they should exhibit a high local variance of gray values. With this in mind, we selected the variance-based segmentation method of Niblack [40]. In this method, the local mean *m* and the local standard deviation *s* are calculated in a circular region around each pixel at indices *i*, *j*. The pixel value is set to one if its gray value satisfies *G(i,j)* > *m* + *s*/2. We tested different radii and found most convincing results for a radius of 7 pixels and consistent results for slightly altered filter radii. Moreover, the square grid of the pixelated images resulted in different response of this filter for lines oriented under different angles. This artifact could be reduced by including all pixels that are intersected by the circle of radius 7 around the central pixel with a weight factor that corresponds to the fraction of pixel area within this circle.

**Fig 2:**
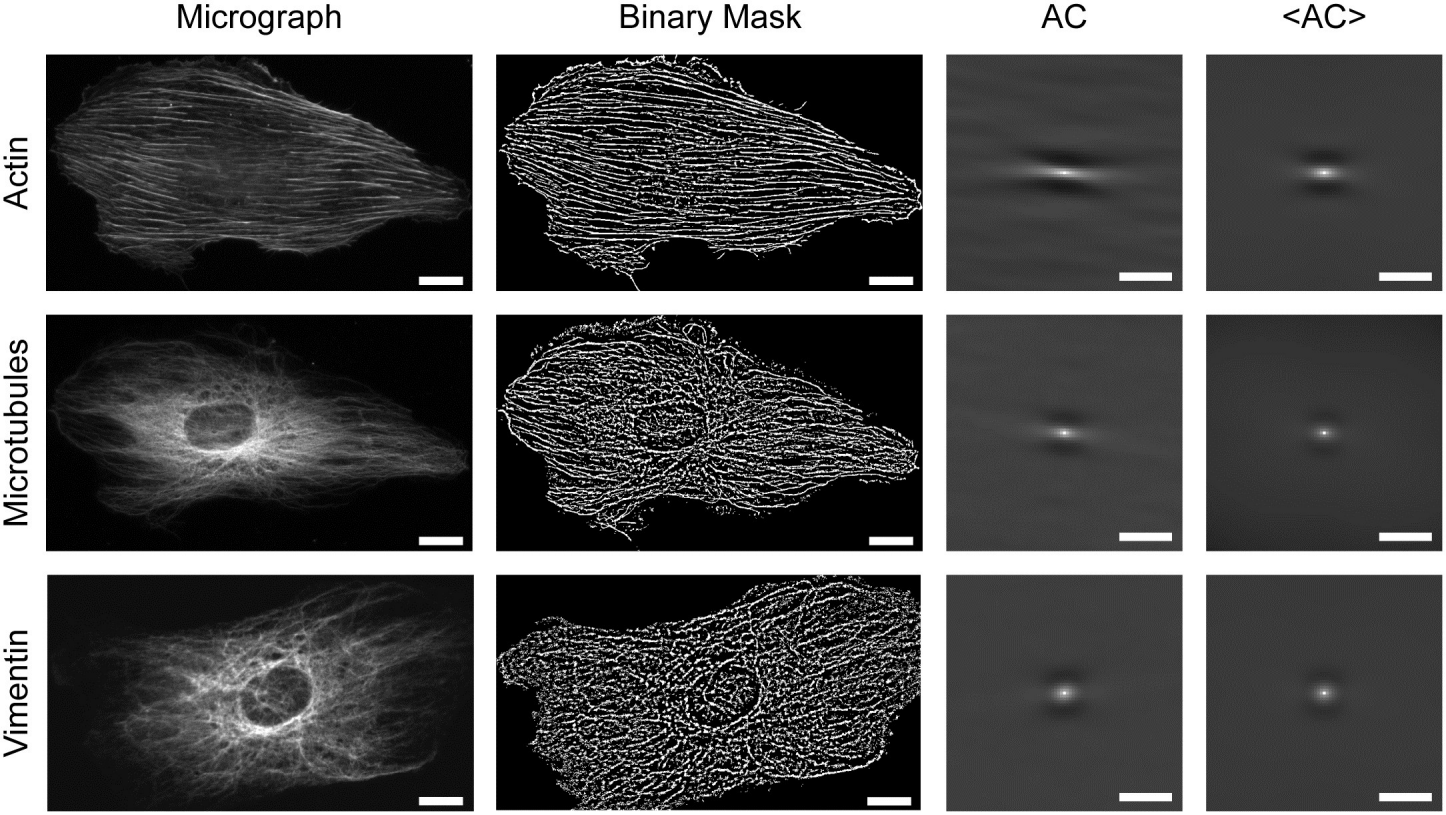
A two-step algorithm consisting of image segmentation and autocorrelation of the resulting binary masks enables calculation of correlograms. Top actin stain, middle microtubules, bottom vimentin. Shown are fluorescence micrographs, the results of segmentation (Binary Mask), the autocorrelograms of the respective cells (AC), and the results of averaging over the whole population (<AC>; 299 cells actin, 688 cells microtubules and 389 cells vimentin). Duration of straining 8 h, stretch direction vertical. Scale bars, 10 μm for micrographs and binary masks, 2 μm for correlograms.

Both masks were combined by a logical AND operation and isolated regions of size one pixel were deleted from the final mask (for examples, see Fig 2). From such *N* by *M* sized binary images autocorrelograms, *AC*, were calculated according to Frykman and Rogon [41].

$$AC\left(i,j\right)=\frac{1}{\left(N-i\right)\left(M-j\right)}\sum_{K=1}^{N-i}\sum_{l=1}^{M-j}I\left(k,l\right)I\left(i+k,j+l\right)$$ (1)

Here the analysis region (*N* by *M* pixels) was the smallest rectangle enclosing the cell. Computation was accelerated substantially by calculating the correlation in Fourier space with ample zero padding to avoid the wrap-around problem [42].

Resulting correlograms of different cells were averaged. It is difficult to determine the statistical uncertainty of the correlation of noisy data, especially if these data sets are of limited length [43, 44]. Therefore we did not apply any weight factors during averaging and determined the statistical uncertainties of the averaged data from the variance between different cells.

Because a binary image, or mask, was correlated, the correlation function *AC(i,j)* has a straightforward interpretation. It is simply the joint probability that one point and another shifted by a vector *(i,j)* are both on the mask. Therefore, it decays slower in fiber directions than in others. Moreover, this algorithm is sensitive to translational order and faithfully indicates multimodal distributions of fibers. Examples are shown in S1 Fig. Nevertheless, especially at longer durations of stretching the vast majority of cells displayed unimodal distributions.

Since autocorrelation is extremely efficient in extracting repetitive features, an influence of the segmentation algorithm on the final correlograms was expected. To test this we produced synthetic data containing realistic noise and applied the above algorithm to them, see S2 Fig. In the correlograms of these synthetic data, direction and length of lines were clearly seen, but their width was obscured by a filter-induced artifact consisting of a dark halo around fibers in the segmented image. Therefore the bright bow-tie shaped structures in the centers of the correlograms in Fig 2 reflect fiber orientation and length distribution but the kidney shaped dark spots above and below are artificial products of the Niblack filter.

### Strain-induced ordering of cytoskeletal systems

**Fig 3:**
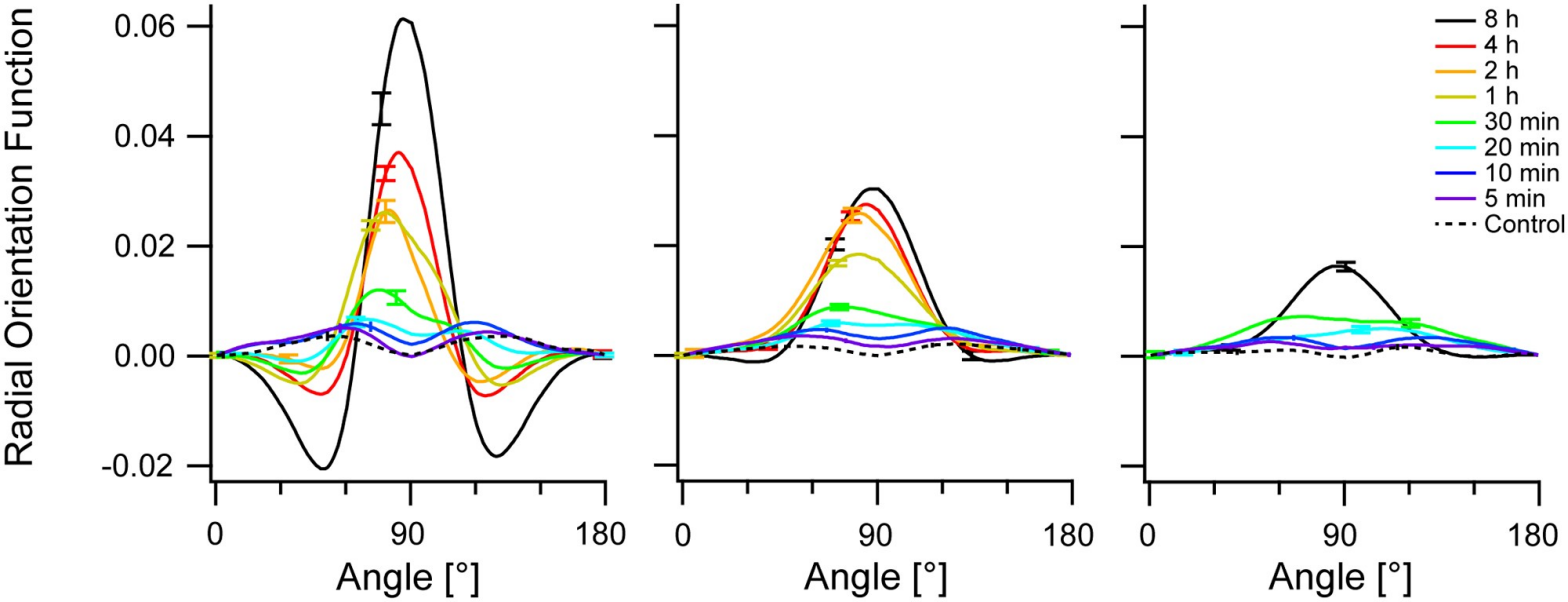
Radial orientation increases with duration of cyclic straining. Shown are radial orientation functions (see text for definition). From left to right: actin, microtubules, and vimentin. Population averages are shown, offset (value at 0°) subtracted. Direction of strain was 0°. Color code depicted at the right and scale shown at the left apply to all graphs. Bars are standard error of mean; for clarity only highest and lowest value at each condition shown. Sample sizes in order from long to short duration: actin 299, 1082, 276, 1958, 553, 597, 502, 386, 415; microtubules 688, 1082, 276, 1958, 973, 1139, 1024, 904, 863; vimentin 389, 420, 542, 522, 518, 448. Note that 4 h, 2 h, and 1 h duration were not measured for vimentin.

With increasing duration of cyclic strain all three cytoskeletal systems ordered substantially, cf. Fig 1. Here, both orientational and translational order increased. Both could be quantified using the autocorrelogram. For the analysis of orientational order we normalized the individual two-dimensional autocorrelograms by the area coverage. Then the individual correlograms were analyzed in a ring around the center (outer radius 21 pixel corresponding to 2.35 μm, inner radius 9 pixel or 1.01 μm, i.e. beyond the size of the Niblack filter kernel) where we determined the average value of the autocorrelation function in 1° wide slices. The result of this calculation is called "radial orientation function" in the following. Radial orientation functions were averaged over all cells investigated at identical conditions. Results are shown in Fig 3. In this averaging step variations from cell to cell were analyzed. Because the values were almost normally distributed, we represent scatter by standard errors of mean. Moreover, as the autocorrelogram is by its construction point symmetric around the center, both half circles were averaged.

This procedure (cf. Fig 3) clearly showed by far the strongest directional order for the actin cytoskeleton. Here, both final order and the kinetics of build-up were highest. Compared to this, microtubules reoriented somewhat less and slower whereas vimentin only reoriented weakly at very long duration of cyclic straining. Because maximum effects were mostly observed at 90° orientation we calculated statistical significance and effect size from the individual values of the radial orientation function at this angle. For details see Materials and methods. Results are given in S1 Dataset. We find statistically significant effects already at 5 min duration for microtubules and vimentin whereas the actin signal reaches clear significance only at 30 min and beyond. Nevertheless, after 8 h effect size is highest in actin, intermediate in microtubules and lowest for vimentin.

From these plots the angle under which the respective cytoskeleton ordered was determined as the angle of highest correlation, that is, the angle under which the absolute maximum of the radial orientation function of the individual cell occurred. It will be called "preferred direction" in the following. As this parameter describes just the preferential direction of the cytoskeletal systems that is also determined by the structure-tensor based approaches (e.g. [8]), we will not discuss the statistics of the preferred directions further beyond mentioning that both algorithms gave similar final conclusions.

**Fig 4:**
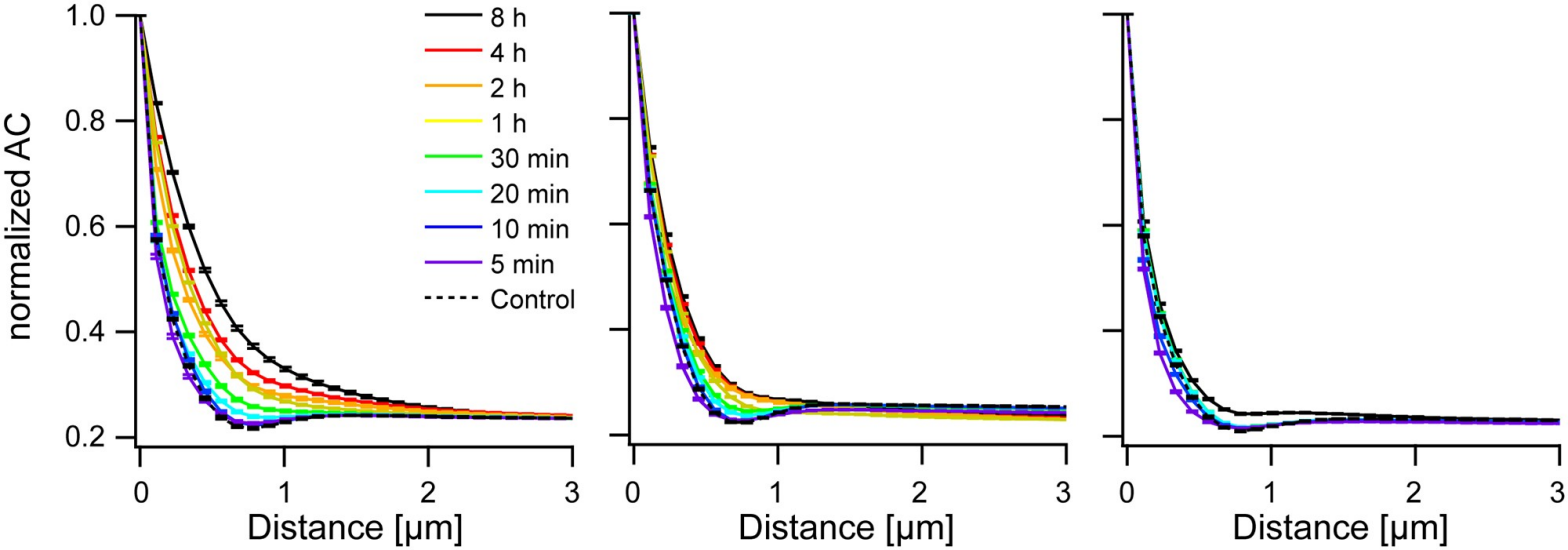
Translational order increases during reorientation. Shown is the evolution of translational order along the angle of highest correlation (the preferred direction) during reorientation. From left to right: actin, microtubules, and vimentin. Correlograms were averaged and normalized by their value at the origin. Bars are standard error of mean. Sample sizes as in Fig 3. Note that 4 h, 2 h, and 1 h duration were not measured for vimentin.

In the averaged autocorrelograms line profiles were drawn along the preferred direction. Again actin reacted strongest to mechanical straining with a pronounced increase in correlation along the preferred direction. Compared to actin, microtubules exhibited markedly less correlation after 8 h of stretching. Moreover, vimentin barely displayed any reaction at all, see Fig 4. Thus, we found similar effects in both types of order analyzed, translational and rotational order.

#### Strain-induced increase of intrinsic cytoskeletal order

The above analysis of population averaged autocorrelograms clearly showed a significant increase in cytoskeletal order upon cyclic straining, most pronounced for actin. Presumably two different effects contribute to this. First, cytoskeletal systems in individual cells change their orientation as a whole with the entire cell (cf. Fig 1), that is, they simply follow the cell body because they are enclosed in its elongated shape. Since without external straining the preferred orientation of a given cell is random and with cyclic straining it reorients towards an externally defined axis, intrinsic cell orientations are progressively aligned with increasing duration of straining. Obviously, even if the order within a cell remained unchanged, this will result in higher and narrower peaks of the averaged correlograms. Second, the cytoskeletal order within each cell, irrespective of the overall cell orientation, might increase with prolonged stretching.

To disentangle these two effects we analyzed our data in a slightly different way: After determining the orientation of the cytoskeleton within a given cell its correlogram was rotated so that the preferred direction was at 90°. Only after this, correlograms of different cells were averaged as before. This alignment procedure removed the effects of angular rotation of the cell body and the cytoskeletal systems with it. Each increase in order seen after this alignment step was solely due to an increase in cytoskeletal order in individual cells. We refer to this type of order as "intrinsic order".

**Fig 5:**
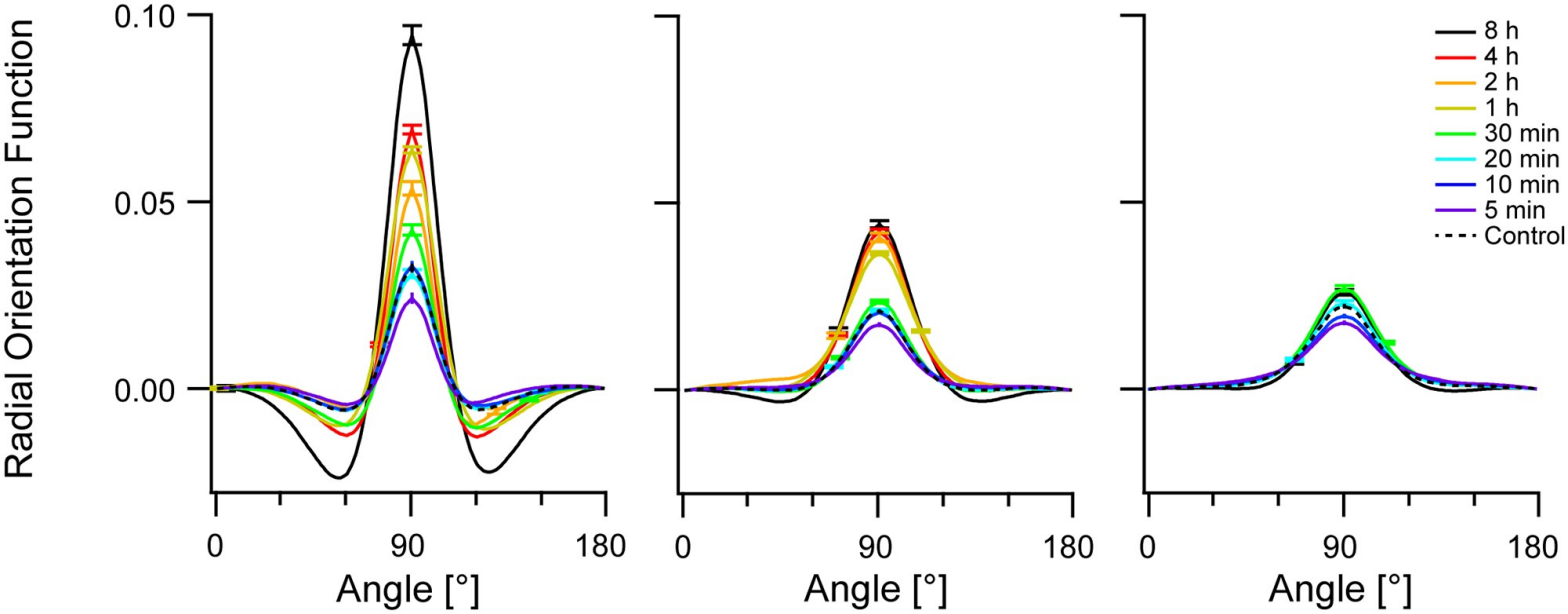
Actin intrinsic orientational order increases strongly with prolonged stretching. Autocorrelograms of individual cells were aligned before calculating the radial orientation functions and averaging them. From left to right: actin, microtubules, and vimentin. Population averages are shown, offset (value at 0°) subtracted. Color code depicted at the right and scale shown at the left apply to all graphs. Bars denote standard error of mean; for clarity, only highest and lowest value shown. Sample sizes are as in Fig 3.

**Fig 6:**
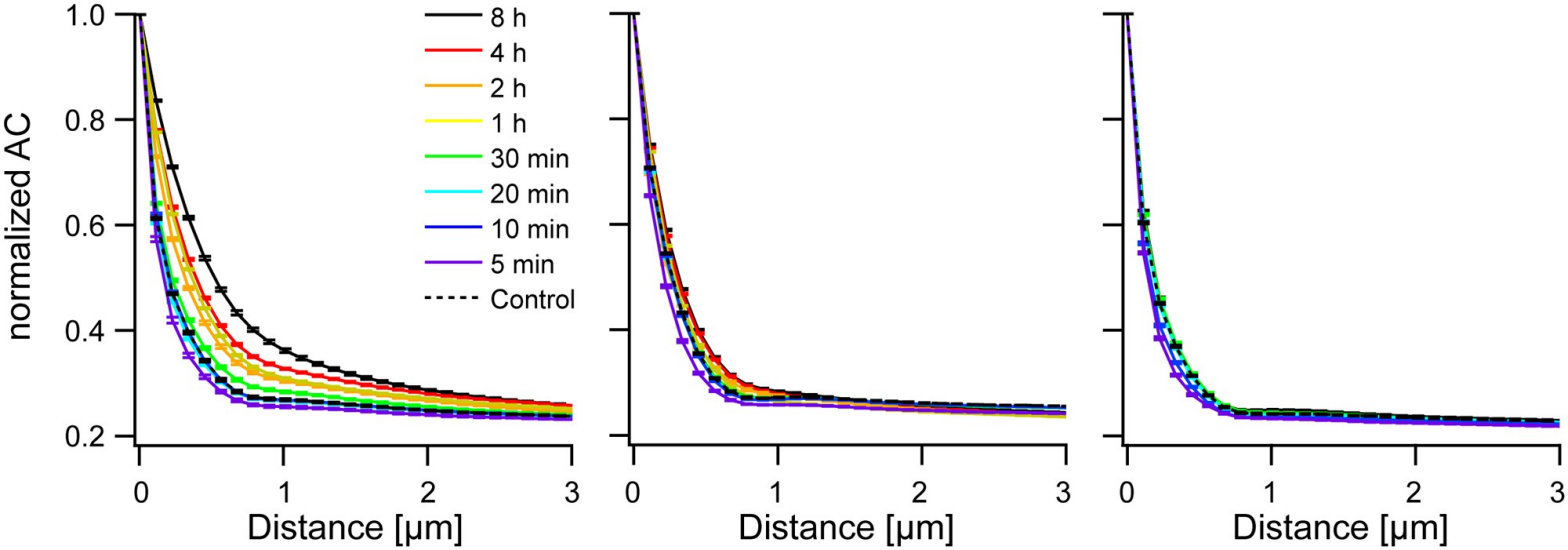
Intrinsic translational order increases during reorientation. From left to right: actin, microtubules, and vimentin. Correlograms were aligned and normalized by their value at the origin before averaging. Shown are values along the direction of shallowest decay. Bars show standard error of mean. Sample sizes are as in Fig 3.

The results of this analysis (Figs 5 and 6) clearly indicated, first, the presence of considerable orientational order within individual cells even before straining and, second, a very substantial increase of orientational (Fig 5) and translational (Fig 6) order of the actin cytoskeleton within each cell as a result of cyclic straining. The microtubule network also showed an appreciable increase of intrinsic order whereas vimentin remained mostly unaffected. Moreover, actin order built up continuously starting from 30 min duration of straining to the very end (8 h). In contrast, microtubule order increased in just one single step between 30 min and 1 h. Statistical significance and effect size are given for the intrinsic radial orientation function (at an angle of 90°) in S2 Dataset. Intriguingly we found for all three cytoskeletons highly significant effects already after 5 min. After this short duration peak values were clearly reduced, as indicated by negative effect sizes (-0.34, -0.32, and -0.45 for actin, microtubules and vimentin, respectively). For longer durations the values of the intrinsic orientational order increased again. This resulted first in return to values similar to the control and, thus, vanishing or lower statistical significance at 10 min and 20 min duration. For longer periods, order was rebuild and increased beyond control values. In vimentin this increase was quite moderate with effect sizes of 0.34 after 30 min and only 0.25 after 8 h, whereas microtubule order increased strongly (effect size 1.3 after 8h) and actin even more so (effect size 2.0 after 8 h). Thus the three cytoskeletal systems responded very differently to cyclic straining. Actin bundles, and a bit less so, microtubules increased their intrinsic radial orientational order as well as their intrinsic translational order. However, intrinsic vimentin order showed only very modest changes.

**Fig 7:**
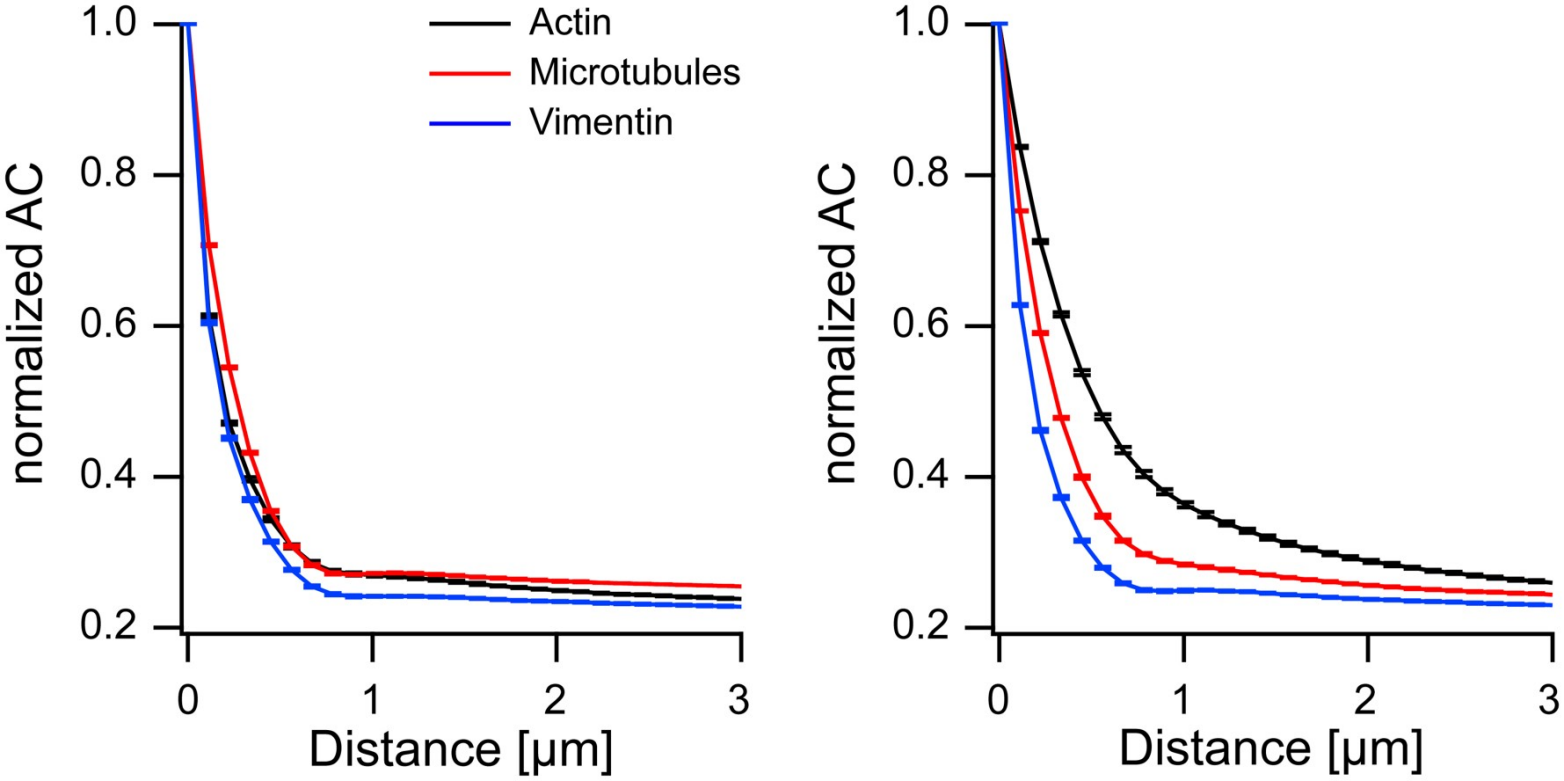
Intrinsic translational order reacts in a graded fashion for the different cytoskeletal systems. Shown are line profiles along the preferred direction in averaged autocorrelograms for control samples (left) and after 8 h of cyclic straining (right).

The different ways in which the different cytoskeletal systems reacted to external straining are clearly seen in a plot of the intrinsic translational order, see Fig 7. Before straining, intrinsic translational order of the three cytoskeletal systems is very similar with slightly lower correlations for vimentin than for the other two systems. After 8 h of straining, however, the intrinsic translational order of actin increased substantially, the one of microtubules only marginally, and vimentin displayed no significant reaction at all.

Taken together, in the overall reaction to external straining, aligning of cell orientations and increasing intrinsic order contribute about equally for actin. For microtubules, intrinsic order still changes significantly but for vimentin this process is only very weak.

### Relation between different cytoskeletal systems

As next step, the interrelation between different cytoskeletal systems was analyzed. Because we were not able to establish an experimental protocol reliably resulting in high quality simultaneous staining of all three cytoskeletal systems, we could analyze only the relation between actin and microtubules and between microtubules and vimentin. In both cases we built on our above procedure for the autocorrelation analysis with the one modification that here two different masks determined for one and the same cell were cross-correlated.

The cross-correlation function, *CC*, of two masks, *I*_*1*_ and *I*_*2*_, is given by

$$CC\left(i,j\right)=\frac{1}{\left(N-i\right)\left(M-j\right)}\sum_{k=1}^{N-i}\sum_{l=1}^{M-j}I_{1}\left(k,l\right)I_{2}\left(i+k,j+l\right)$$ (2)

where *N* and *M* are the dimensions of the smallest rectangle enclosing the masks.

**Fig 8:**
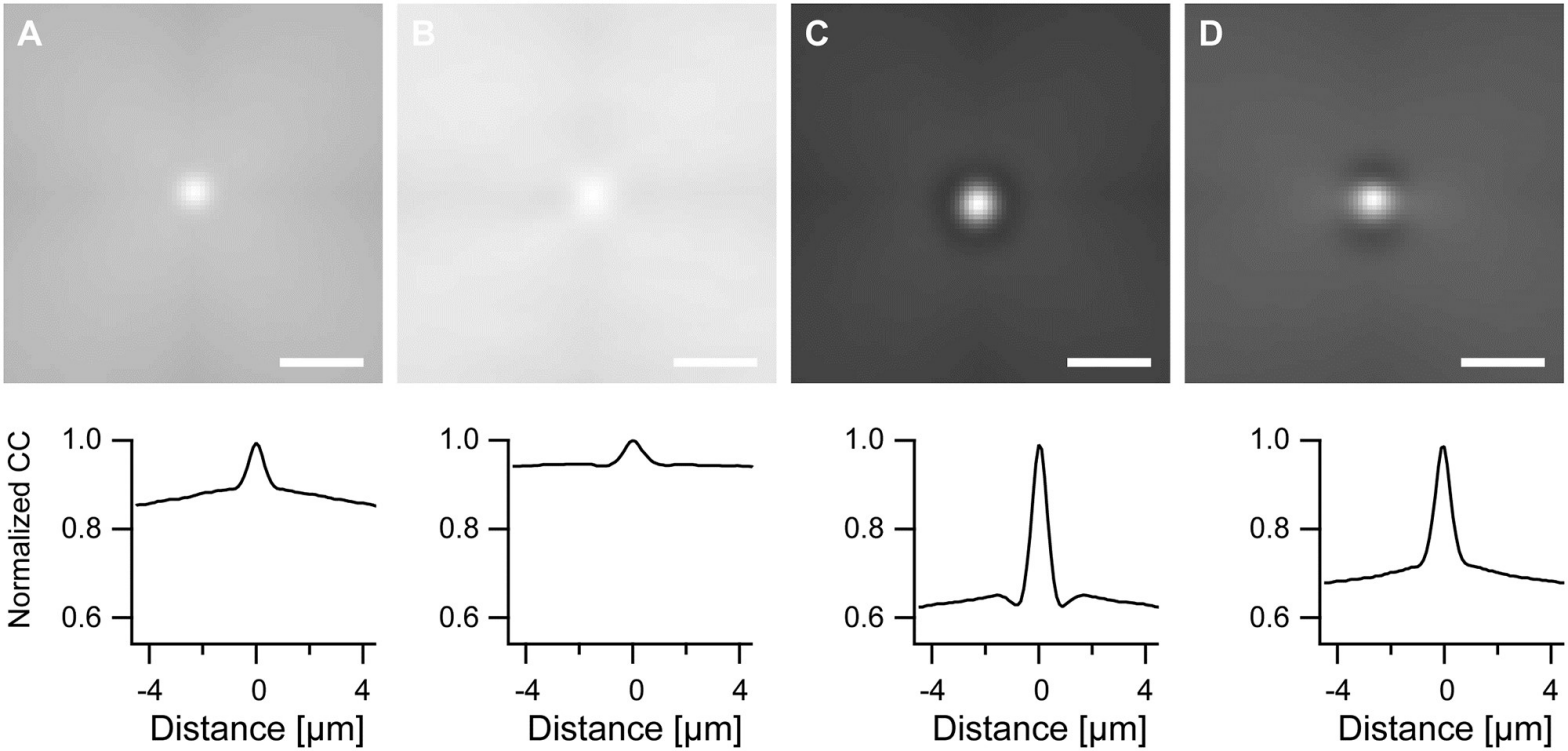
Stretching reduces resemblance between cytoskeletal systems. Shown are normalized average cross-correlograms. For all correlograms: maximum set to 1.0; gray scale ranges from 0.5 (black) to 1.0 (white). Scale bars, 2 μm. In the lower row intensities along horizontal lines through the maxima are displayed. A) Actin versus microtubules, unstrained (415 cells, maximum cross-correlation 0.035); B) actin versus microtubules, 8 h stretch (299 cells, maximum 0.0312); C) vimentin versus microtubules, unstrained (448 cells, maximum 0.0481); and D) vimentin versus microtubules, 8 h stretch (389 cells, maximum 0.0432).

Cross-correlograms, Fig 8, exhibited a central peak residing over a background whose intensity varied only on extremely large length scales. This central peak indicated to which extent the different cytoskeletal systems overlapped. The smooth background originated from the fact that we cross-correlated rectangular regions enclosing the respective cells where the out-of-cell regions were black and most often in the periphery of the rectangle. With increasing displacements the still overlapping regions of the rectangles contained an increasing fraction of out-of-cell areas with the consequence of reduced values of the correlograms. Because even for the longest duration of stretching cross-correlograms indicated no preferential orientation beyond the halo artifact (cf. Fig 8), we focused here on a comparison between control (no stretch) and 8 h duration of stretch.

We found for the cross-correlation of the actin mask with the microtubule mask a peak height (as measured from the background level) of 3.9 10^−3^ residing on a background of 31 10^−3^ without stretch and 1.7 10^−3^ (background 29 10^−3^) after 8 h stretch. For vimentin with microtubules these values were 16 10^−3^ (background 31 10^−3^) without and 12 10^−3^ (background 31 10^−3^) after 8 h stretch. From these measurements it is very obvious that the microtubule cytoskeleton and the vimentin cytoskeleton display a much higher cross-correlation than the microtubule and actin cytoskeleton. Here, again, actin is set apart from the other two cytoskeletal systems. Notably, prolonged cyclic stretch reduced both cross-correlations.

In principle, even two randomly distributed and unrelated networks will show some cross-correlation if they are contained in the same delimiting shape. Moreover, microtubule and vimentin density decreased from the center to the periphery of the cell. For these reasons it was not clear if the observed cross-correlations were significant or just the result of chance. To answer this question, we cross-correlated cytoskeletal systems (actin versus microtubules and vimentin versus microtubules) contained in different, randomly selected cells with identical stretch history. Obviously, cytoskeletal systems from different cells cannot exhibit a biologically meaningful resemblance on short length scales thus all cross-correlations observed here are not biologically meaningful.

**Fig 9:**
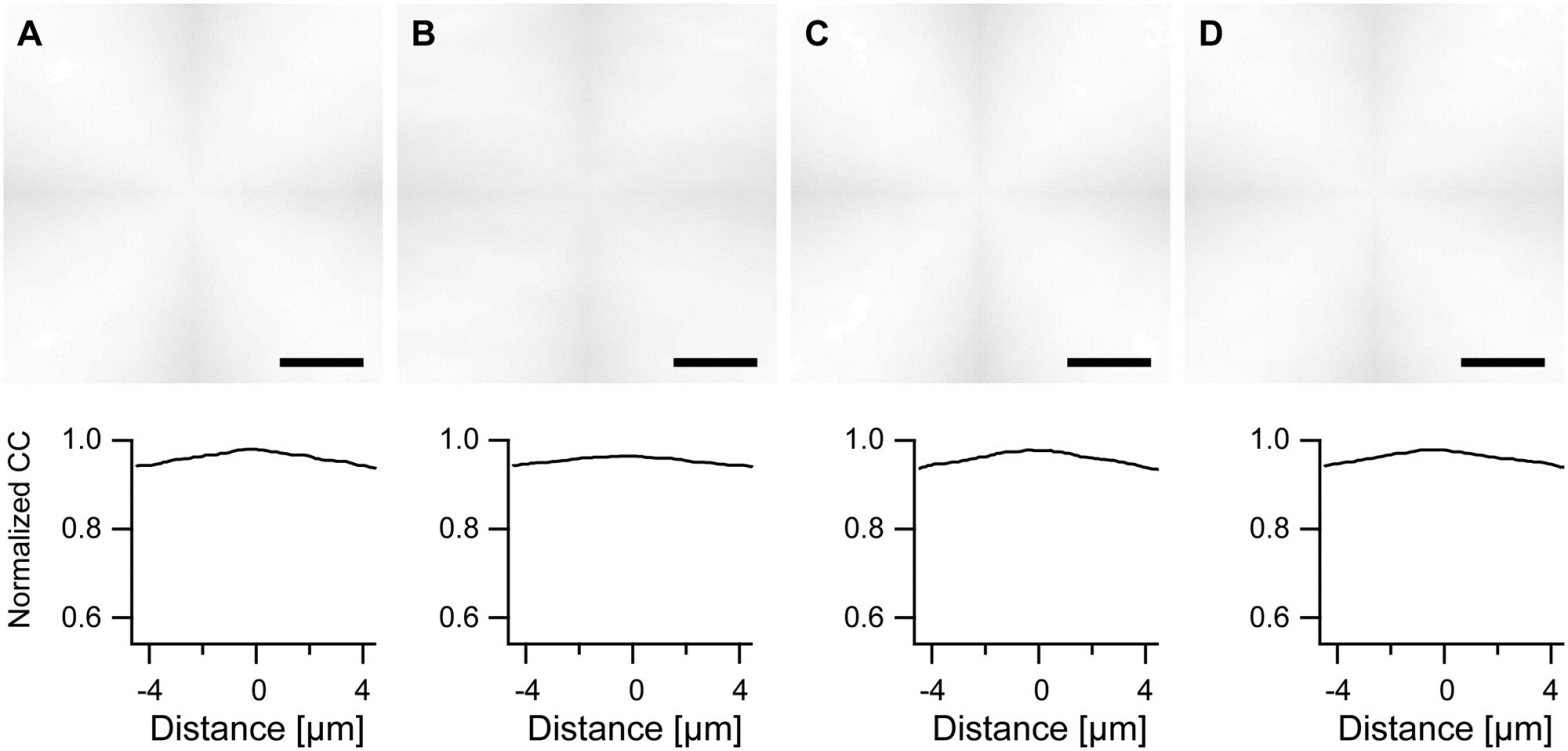
Central peaks in cross-correlograms indicate overlap of cytoskeletal systems. Shown are normalized average cross-correlograms between cytoskeletal systems taken from different, randomly selected cells calculated within the largest circle fitting into both cells. Therefore only artificial correlations can occur. For all correlograms: maximum set to 1.0; gray scale ranges from 0.5 (black) to 1.0 (white). Scale bars, 2 μm. In the lower row intensities along horizontal lines through the maxima are displayed. A) Actin versus microtubules, unstrained (415 cells, maximum cross-correlation 0.0517); B) actin versus microtubules, 8 h stretch (299 cells, maximum 0.0436); C) vimentin versus microtubules, unstrained (448 cells, maximum 0.0517); and D) vimentin versus microtubules, 8 h stretch (389 cells, maximum 0.0504). Note the absence of central peaks (cf. Fig 8).

Because the two unrelated cytoskeletons originating from different cells filled differently shaped regions, the analysis had to be slightly modified. Before, we used the information of the full area of the cell. Here, analysis was limited to the largest circle that fitted into both cells. All other data were ignored. Specifically, we cross-correlated the binary mask representing the actin cytoskeleton in one cell with that of the microtubules in another, randomly chosen cell within the largest circle that could be placed into both cells. This was repeated for all cells and the resulting cross-correlograms averaged. The same procedure was used for controls and stretch duration of 8 h as well as for calculation of cross-correlograms of vimentin with microtubules. The resulting cross-correlograms (Fig 9) exhibited no structure besides a slight cross-shaped artifact. This originated from the fact that to maintain the run time efficiency of the fast Fourier transform, cross-correlograms were calculated in square-shaped regions enclosing the circular region from which data were used. Therefore in Eq 2 the overlap area of two circles shifted by the vector *(i,j)* should have been used for normalization instead of the factor *(N-i)(M-j)*.

The absence of central peaks in these cross-correlograms showed that these peaks specifically indicated overlap of different cytoskeletal systems. They were not due to indirect effects like spatial variation of cytoskeletal density or similarity of cell shapes.

**Fig 10:**
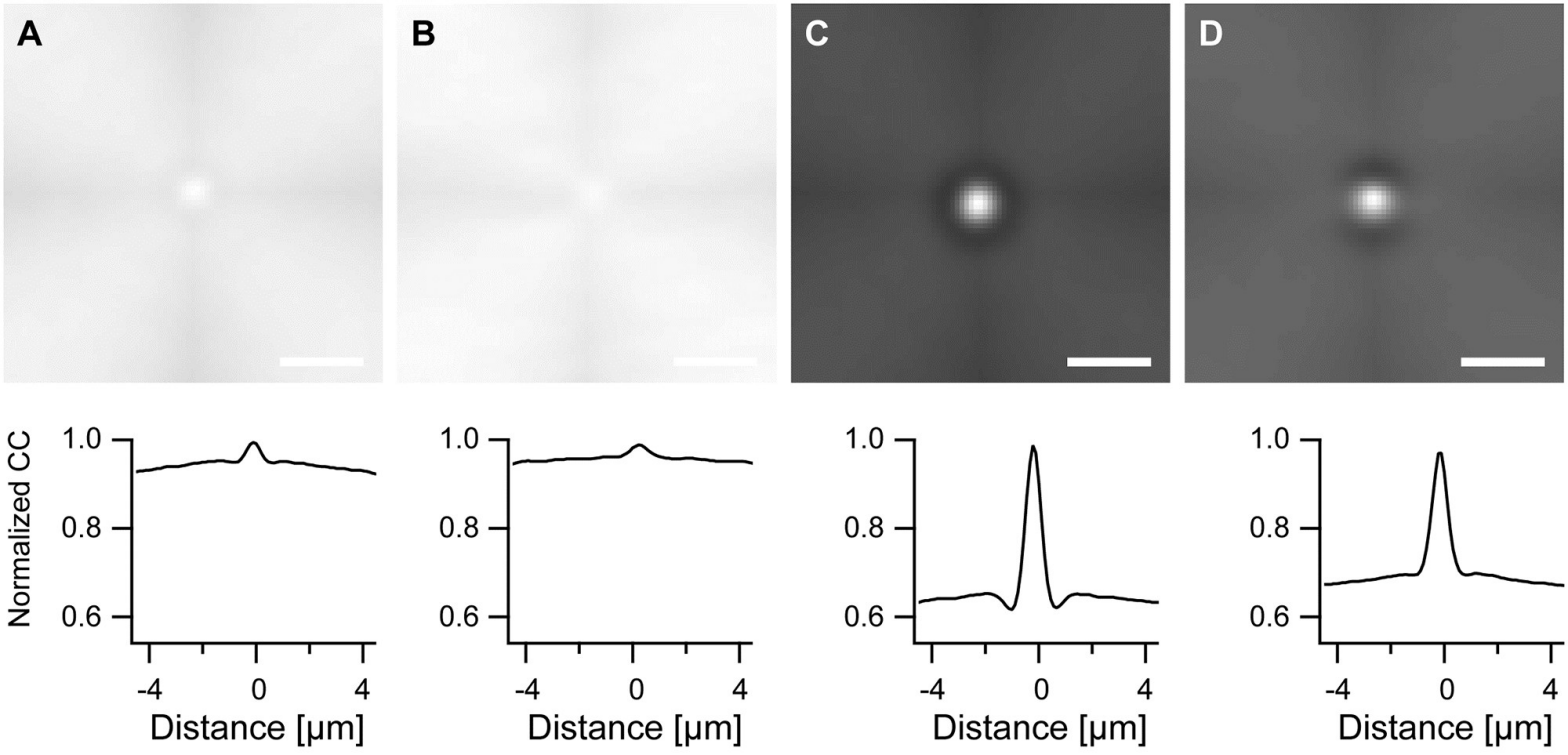
Restricting cross-correlation analysis to the central region of the cell, that is the largest circle fitting into it, gives similar results as compared to the full cell analysis. Shown are normalized average cross-correlograms taken from the largest circle fitting into the cell. For all correlograms: maximum set to 1.0; gray scale ranges from 0.5 (black) to 1.0 (white). Scale bars, 2 μm. In the lower row intensities along horizontal lines through the maxima are displayed. A) Actin versus microtubules, unstrained (415 cells, maximum cross-correlation 0.0527); B) actin versus microtubules, 8 h stretch (299 cells, maximum 0.0432); C) vimentin versus microtubules, unstrained (448 cells, maximum 0.0481); and D) vimentin versus microtubules, 8 h stretch (389 cells, maximum 0.0432).

Yet the two cross-correlation analyses differed in the cell areas used for calculation. For comparison of different cytoskeletal systems within one cell (Fig 8) calculation was done in the full cell area whereas for the significance test (Fig 9) circular areas were used only. Therefore we had to check if this different region of analysis would alter the findings obtained for the full cell (Fig 8). This spatially restricted cross-correlation analysis of different cytoskeletal systems within the same cells gave similar main findings as before, compare Figs 8 and 10. Still some intriguing variations were seen in quantitative details.

In detail, for the cross-correlation of actin with microtubules we found a small peak of a mere 2.4 10^−3^ (as measured from the background level) residing on a background of 50 10^−3^ without stretch. The respective values were 1.4 10^−3^ and 41 10^−3^ after 8 h cyclic stretching. Cross-correlation of vimentin with microtubules resulted in a pronounced peak of 26 10^−3^ height residing on a background of 50 10^−3^ without stretch and 20 10^−3^ peak height on a 49 10^−3^ background after 8 h cyclic stretching. Compared to the cross-correlation using the full cell shape we found higher cross-correlations for vimentin with microtubules and lower values for actin with microtubules. Because the procedure of using only the largest circle fitting into the cells removes the most peripheral regions from the analysis, this result implied a spatial dependence of the cross-correlations. The cross-correlations between actin and microtubules were higher at the periphery of the cell than in the center while the opposite held for the cross-correlations of vimentin and microtubules. Again, prolonged stretch reduced the cross-correlations between both cytoskeletal systems.

### Effects of microtubule depolymerization

**Fig 11:**
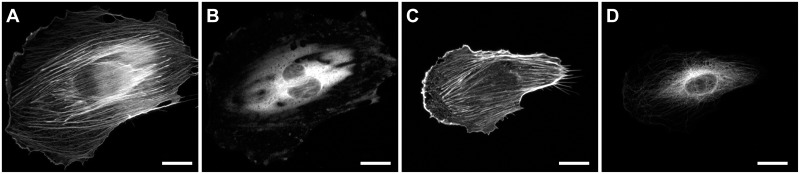
Depolymerization of microtubules leaves actin response intact. Shown are exemplary micrographs of cells treated with 10 μM nocodazole (A, B) and control cells treated with equal amounts of DMSO (C, D). Staining for actin (A, C) and tubulin (B, D). All cells: duration of straining 20 min, stretch direction vertical. Scale bars, 20 μm.

For a further exploration of the interdependence between the different cytoskeletal systems we disrupted the microtubule cytoskeleton with nocodazole. We did not attempt to investigate cells that were treated with latrunculin to depolymerize the actin cytoskeleton because Zielinski et al. already reported for such cells a complete breakdown of structure upon stretching [36]. In our experiments duration of stretching was limited to 30 minutes since at longer periods cells were visibly harmed. Moreover, control experiments where cells were treated with DMSO alone showed less well developed microtubule cytoskeletons than cells that were not exposed to this solvent. Typical results are shown in Fig 11.

**Fig 12:**
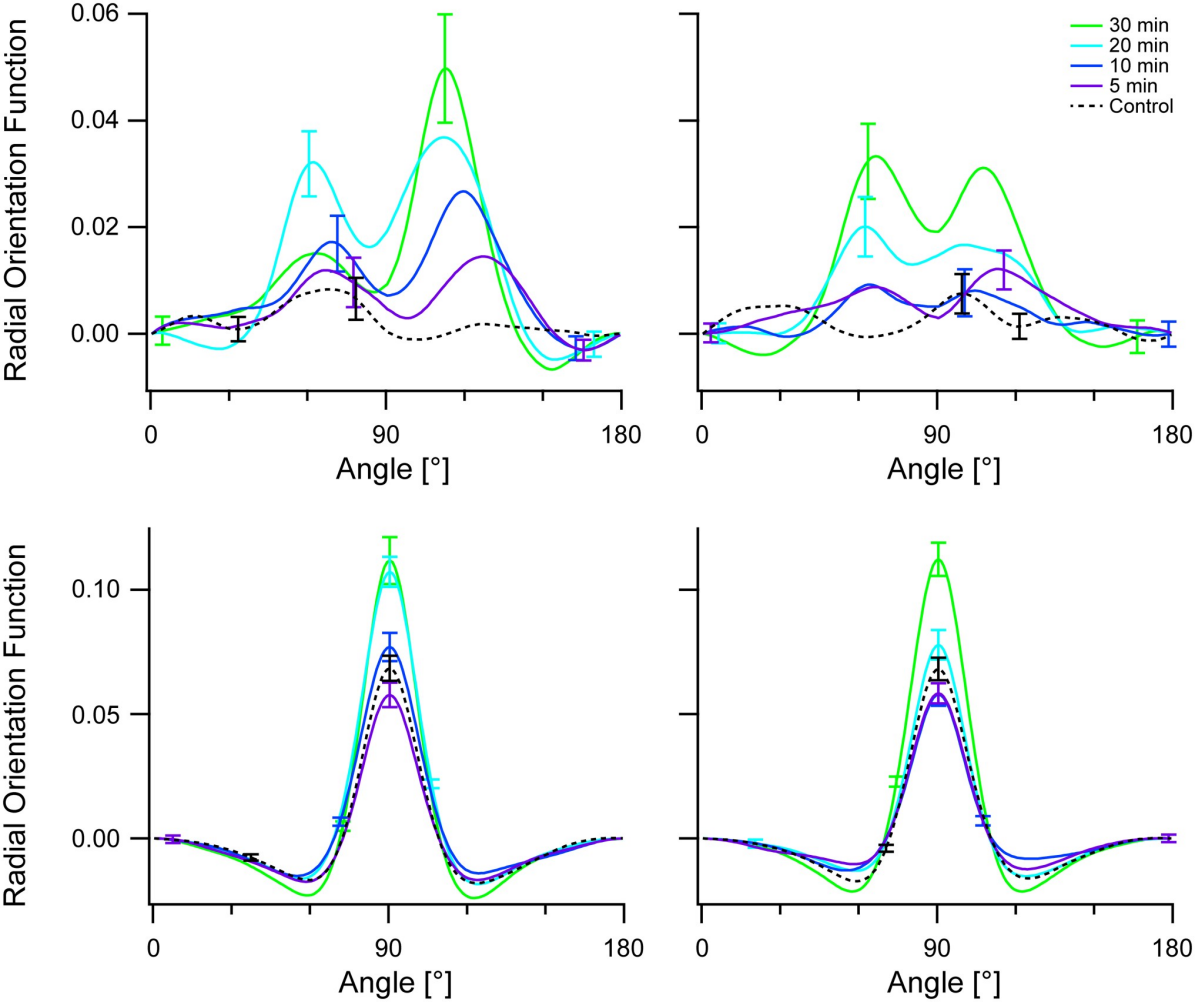
Depolymerization of microtubules leaves actin orientational order intact. Shown are radial orientation functions (top row, compare Fig 3) and intrinsic radial orientation obtained by aligning autocorrelograms before average (bottom row, compare Fig 5) of cells treated with 10 μM nocodazole (left column) or DMSO alone (right column). Population averages are shown, offset (value at 0°) subtracted. Direction of strain was 0°. Bars are standard error of mean; for clarity only highest and lowest value at each condition shown. Sample sizes were for nocodazole treatment 58, 98, 87, 52, and 111; for DMSO alone 83, 88, 76, 95, and 107, both in order from long to short duration.

From these data we extracted radial orientation functions of the actin cytoskeleton (compare Fig 3 for untreated cells) and intrinsic orientational order (compare Fig 5 for untreated cells). The results are displayed in Fig 12. They clearly showed that the build-up of order was very similar in both sample types. In other words, the absence of the microtubule network did not impede the build-up of order in the actin cytoskeleton. In fact, the data even showed a tendency for a slightly faster kinetics that is also reflected in the statistical comparison of both sets of data (S3 Dataset). Here we found significantly higher intrinsic order for nocodazole treated cells after 10 min and 20 min straining (effect sizes of 0.44 and 0.52, respectively), at all other conditions intrinsic order did not significantly depend on nocodazole treatment. No significant differences at all were found in the radial orientation function, that is without alignment of autocorrelograms before averaging. Overall these data and our analyses showed that reorientation and build-up of intrinsic order in the actin cytoskeleton did not depend on the presence of an intact microtubule network.

## Discussion

In this project we established a two-step algorithm to quantify cytoskeletal order from immunofluorescence micrographs. Micrographs were first converted into binary masks representing cytoskeletal structures. Subsequently correlations in these masks were analyzed. For the identification of cytoskeletal structures we reasoned that they should exhibit high intensity variation on short length scales, at least in one direction, because they are formed by thin fibers. Therefore we used the variance of gray values in a circular region of approximately 0.8 μm radius as selection criterion. Obviously, this type of segmentation will not discriminate against other small objects like small debris of fluorescent label or even noise in the image. Because we were aiming for a conceptually simple algorithm with transparent functioning and virtually no user interaction, we decided to restrict all further reduction of artifacts to the deletion of isolated regions of size one pixel as these cannot form parts of fibrous structures. All further steps would have entailed many, very specific criterions that would have to be tailored for each type of cytoskeleton in each specific cell type. By this we would have lost the possibility to compare different cytoskeletal systems. This contribution of non-cytoskeletal origin could be tolerated because the subsequent correlation step is extremely efficient in extracting repetitive features even from very noisy data. Thus all point-like, roundish or irreproducible features just contributed to the overall noise-floor of the final correlogram. However, the presence of non-fibrous structures in the masks was certainly diminishing the values of the correlation functions.

These correlograms clearly showed directions of fiber orientations, see Fig 2. These orientations can be also determined by texture analysis as presented e.g. in [8]. However, correlograms contain much more information on cytoskeletal order than just this one angle. First of all, the decay of the correlation along this direction (see Fig 4) was determined. Here, especially averages over cell populations strained for long durations were instructive. As expected, the lateral correlation was highest for actin and least for vimentin. However, correlations were relatively short ranged, with actin order reaching out to about 3 μm, vimentin to only about 1 μm and microtubules in the middle (cf. Fig 4). While this appeared surprising, especially for actin (cf. Fig 2), a closer inspection of micrographs showed many shorter filaments and other small structures besides very prominent, straight and long stress fibers. Moreover, the spread in orientation of the different stress fibers also contributed to the decay of the correlation function.

Especially micrographs of the actin cytoskeleton often showed an obvious preferential orientation even for unstrained cells that was lost during averaging because the orientations of different cells were unrelated. Thus even without stretching there was cytoskeletal order and two different processes, namely alignment of cells and increasing order within each cell, could contribute to the ordering phenomena observed. To answer this question we analyzed the average intrinsic order by aligning the individual correlograms before they were averaged. Indeed, the results of this procedure were consistent with the above analysis of averaged correlograms and showed the main findings with even higher clarity. Both, in radial and transverse direction, actin displayed the highest order and reacted most to cyclic straining, cf. Figs 5–7. The increase of order took place from 30 min duration to 8 h, the longest duration tested. In contrast, microtubules still reacted to cyclic strain but to a much lower degree and with a different time course. Here the buildup of order occurred in one step from 30 min duration to 1 h. Surprisingly, intrinsic vimentin order seemed almost identical for control cells and after 8 h of stretching. These differences in kinetics are most likely at least in part due to the influence of strain on protein synthesis and turnover.

An intriguing observation is the decay of order after short straining of 5 min duration followed by a slower rebuilding of order. This effect was clearly seen in aligned correlograms with high statistical significance and moderate effect sizes of about -30%. This partial disorganization of the actin cytoskeleton is most likely related to the stretch induced cytoskeletal fluidization found by the Fredberg group from the mechanics of single cells undergoing stretch [45, 46]. Surprisingly similar effects were seen in all three cytoskeletons.

Thus while the initial response of the three cytoskeletal systems to stretching appears similar, the long time response is clearly different: Actin reacts most, microtubules less so and the intermediate filament vimentin barely. This is in line with the biological state of endothelial cells plated on a fibronectin coated substrate without contact to neighbors. In this situation these cells adhere mostly via focal adhesions to the extracellular matrix. In these adhesion structures the actin cytoskeleton is linked via a plethora of connecting proteins to the intracellular domains of integrins that, in turn, are bound to the extracellular matrix [47–49]. Within the cell focal adhesions are connected by stress fibers that are thick bundles of actin fibers containing also cross-linking and motor proteins [50–52]. Therefore mechanical strain of the substrate is directly acting on connected actin fibers. This is most likely the cause for the prominent reaction of actin to external stretching observed here. In addition, the almost complete breakdown of cytoskeletal order upon cyclic stretch of latrunculin treated cells observed by Zielinski et al [36] further highlights the dominant role of the actin cytoskeleton in cellular response to cyclic substrate strain. Thus at least for endothelial cells at moderate external strain, the actin cytoskeleton is the structure that has to bear the load.

Microtubules are well known to interact transiently with focal adhesions. As a result of these interactions focal adhesion turn-over is increased and microtubule dynamics is altered [53–55]. However, this type of contact is short lived and most frequently associated with destabilizing of focal adhesions whereas stabilization of microtubules by focal adhesions is rarely mentioned [56]. Thus the motion of focal adhesions with the substrate is expected to only indirectly influence microtubules. In contrast, vimentin has been described to associate at least with a sizeable fraction of matrix adhesions in microvascular endothelial cells [57, 58] and fibroblasts [59]. Thus vimentin is expected to be exposed to substrate strain via the motion of cellular adhesions with the substrate whereas microtubules are only indirectly affected. Therefore it is slightly surprising that in our experiments microtubules showed a significant response to external stretching while vimentin barely reacted. A possible explanation for this counterintuitive behavior might be the extreme flexibility of the vimentin system that is very disordered and consists of extremely flexible fibers. It might be that the strain applied here is simply not large enough to induce vimentin reorganization.

Up to now only direct linkages of the individual cytoskeletal systems to substrate stretch have been discussed. However, numerous proteins have been described to directly or indirectly cross-link two of the three cytoskeletal systems. On one hand such molecules may serve to transmit mechanical strain from one cytoskeleton to another. On the other hand, their action should result in spatial correlations between the different cytoskeletal systems. These were studied here by cross-correlation, cf. Figs 8–10. This analysis revealed a strong linkage of microtubules with vimentin while actin and microtubules displayed very weak correlations. The latter fact is in line with literature [14, 16] and our results on nocodazole treated cells (cf. Figs 11 and 12) where almost identical behavior of the actin cytoskeleton for cells with intact and cells with depolymerized microtubules was found.

The weak correlations between actin and microtubules are slightly surprising because a coupling of microtubule and actin movements was described in locomoting epithelial cells [60]. Moreover, many interconnecting molecules have been described, for a recent review see [61]. For example, members of the formin protein family exhibit both actin and microtubule binding domains [61] and a corresponding *in vivo* function has been established for the Drosophila formin Cappuccino [62]. However, formins bind mostly at the plus end of microtubules [63]. Therefore, these molecules can cause only very localized correlations between actin and microtubules that most likely are not sufficiently frequent to show up in our analysis that is based on all fibers at all locations in the cell.

Remarkable in this context is also that many more proteins mediate interactions between microtubule ends and actin or actin-rich structures. An example is ACF7 (actin crosslinking family 7) also called MACF1 (microtubule and actin crosslinking factor 1) that is essential for connecting microtubules and actin in peripheral regions of the cell [64, 65]. A more indirect connection between microtubule ends and actin fibers is provided by G2L1 and G2L2, members of the growth-arrest-specific 2 (GAS2) protein family. These molecules connect actin stress fibers and microtubule end binding proteins. Therefore the consequences of this indirect interaction are most conspicuous in peripheral regions of the cell [66]. Thus more interaction between actin fibers and microtubules is expected in peripheral regions of the cell. This is in line with our finding that exclusion of peripheral regions of the cells from the cross-correlation analysis resulted in reduced correlation between actin and microtubules (compare Figs 8 and 10).

In view of the molecular links between actin and microtubules discussed above one would also expect a relevant influence of microtubules on the actin cytoskeleton. However, no such effect was found in our experiments on nocodazole treated cells (cf. Figs 11 and 12). We believe this to be a consequence of kinetics, as especially the indirect interaction via microtubule end binding proteins cannot act faster than the growth kinetics of microtubules in cells. Unfortunately we could not test this hypothesis because nocodazole induced cell damage already after approximately 1 h incubation.

The high cross-correlation between vimentin and microtubules is also consistent with insight from cell biology, for a review see [67]. Vimentin filaments were shown to associate with detyrosinated microtubules that form a small and stable subset of the microtubule network [68]. Beyond this, a more general microtubule-vimentin cross-linking activity was shown for plectin [69]. The dependence of vimentin filament coalignment with microtubules on the motor protein kinesin is known since a quarter of a century [70]. Later life cell microscopy studies clearly showed that vimentin filaments are formed from very small precursors, termed "dots" that fuse to still short filaments, called "squiggles", which are finally fusing to the ends of growing filaments. These precursors are transported along microtubules by the microtubule attached motor proteins kinesin [71] and dynein [72, 73]. Recent work along these lines demonstrated that remodeling of vimentin filaments occurs via severing of old filaments and reannealing to other filaments [74]. Therefore active transport of short vimentin filaments along microtubules is decisive not only for the growth of new filaments but also for a permanent remodeling of the whole network [74]. Besides being transported bidirectionally along microtubules by kinesin and dynein, short vimentin filaments are frequently trapped by a strong association with filamentous actin [75]. Therefore the vimentin network is shaped by both microtubules and actin fibers with microtubules having the dominant effect. Surprisingly, the cross-correlation between microtubules and vimentin was increased by exclusion of peripheral cell regions from analysis, compare Figs 8 and 9. This spatial distribution of correlation has yet to be explained and requires further study.

We observed that prolonged cyclic stretching reduced the cross-correlations of actin with microtubules and of vimentin with microtubules, see Fig 8 or Fig 9. In view of the fact that the actin cytoskeleton and, to a lesser degree, microtubules as well ordered upon stretching, cf. Figs 3–6, this was a major surprise. This finding of stretch-induced decoupling of cytoskeletal systems might be caused by the fact that several molecular mechanisms causing co-alignment are not due to direct static cross-linking of filaments but are of dynamical nature and act mostly during the development of the individual cytoskeletal systems. If this is indeed the major contribution to the reduction of cross-correlation upon prolonged cyclic stretch, it implies that assembled cytoskeletal structures reorient mostly as a whole with only a limited amount of molecular turnover. Moreover, at least some processes causing the ordering of actin seem to act within this cytoskeletal system alone with little coupling to others. This is highlighted by the fact that actin reorients and orders almost identically in cells with and without microtubules (cf Figs 11 and 12 as well as [14, 16]). These hypotheses could be tested for example by transient knock-down of key cross-linkers or signaling molecules in a similar analysis as described here.

To summarize our findings, the actin-based cytoskeleton is the cellular structure that reacts most prominently to external stretch. The underlying mechanosensory mechanisms are most likely affected by molecules that are mechanically connected more or less directly to the actin cytoskeleton. Clearly, the mechanisms by which external stretch controls order within cytoskeletal systems and the interrelation between different cytoskeletal systems deserve future study and will likely yield more surprises.

## Supporting information

S1 Fig Autocorrelograms reflect translational order of fibrous structures. Shown are actin cytoskeletons and correlograms of control cells (no straining) exhibiting bimodal (left pair) or trimodal (right pair) order of filaments. Scale bars, 10 μm for micrographs, 2 μm for correlograms. Logarithmic lookup table for correlograms.
(TIF)

S2 Fig Analysis of synthetic data shows that orientation and length of fibrous structures can be read from autocorrelograms. Hundred rectangles (5 by 200 pixel) oriented under 34° to the vertical axis with realistic intensity noise added (top left) were segmented (top middle) and autocorrelated (top right, only central 201 x 201 pixels are shown). Line profiles of the correlogram along (bottom left) and normal (bottom right) to the feature orientation are also shown. Note the dark halos around lines and spot-noise in the segmentation. These were a filter-induced artifact and caused a pronounced depression of the correlogram in direction perpendicular to the lines. Fiber length was encoded in the slope of the linear decay of the correlogram along fiber direction.
(TIF)

S1 Dataset Statistical significances (KS test, upper table) and effect sizes (see Materials and methods, lower table) for radial orientation functions (Fig 3) of actin, microtubules and vimentin at an angle of 90° towards stretch. Sample sizes are given in Fig 3 caption.
(XLS)

S2 Dataset Statistical significances (KS test, upper table) and effect sizes (see Materials and methods, lower table) for intrinsic radial orientation functions (Fig 5) of actin, microtubules and vimentin at an angle of 90° towards stretch. Sample sizes are given in Fig 3 caption.
(XLS)

S3 Dataset Statistical significances (KS test) and effect sizes (see Materials and methods) for a comparison of radial orientation functions of the actin cytoskeleton (values at 90°, see Fig 12) of cells treated with nocodazole and control cells treated with DMSO alone. Moreover, same analysis for intrinsic radial orientation of actin, i.e., alignment of correlograms before averaging.
(XLSX)

## References

[1] Chuang JS, Zemljic-Harpf A, Ross RS, Frank LR, McCulloch AD, Omens JH. Determination of three-dimensional ventricular strain distributions in gene-targeted mice using tagged MRI. Magnetic Resonance in Medicine. 2010;64:1281–8. 10.1002/mrm.22547 20981782 PMC3272305

[2] Roan E, Waters CM. What do we know about mechanical strain in lung alveoli? American Journal of Physiology Lung Molecular Physiology. 2011;301:L625–35. 10.1152/ajplung.00105.2011 PMC3213982 21873445

[3] Hadid A, Epstein Y, Shabshin N, Gefen A. Modeling mechanical strains and stresses in soft tissues of the shoulder during load carriage based on load bearing open MRI. Journal of Applied Physiology. 2012;112:597–606. 10.1152/japplphysiol.00990.2011 22134690

[4] Humphrey JD. Vascular adaptation and mechanical homeostasis at tissue, cellular and sub-cellular levels. Cell Biochem Biophys. 2008;50:53–78. 10.1007/s12013-007-9002-3 18209957

[5] Vonk-Noordegraaf A, Haddad F, Chin KM, Forfia PR, Kawut SM, Lumens J, et al Right heart adaptation to pulmonary arterial hypertension. Journal of the American College of Cardiology. 2013;62:D22–D33. 10.1016/j.jacc.2013.10.027 24355638

[6] Buck RC. Reorientation response of cells to repeated stretch and recoil of the substratum. Experimental Cell Research. 1980;127:470–4. 7379874 10.1016/0014-4827(80)90456-5

[7] Dartsch PC, Betz E. Response of cultured endothelial cells to mechanical stimulation. Basic Research in Cardiology. 1989;84:268–81. 2764859 10.1007/BF01907974

[8] Faust U, Hampe N, Rubner W, Kirchgessner N, Safran S, Hoffmann B, et al Cyclic Stress at mHz Frequencies Aligns Fibroblasts in Direction of Zero Strain. Plos One. 2011;6(12). 10.1371/journal.pone.0028963 22194961 PMC3241701

[9] Hayakawa K, Sato N, Obinata T. Dynamic reorientation of cultured cells and stress fibers under mechanical stress from periodic stretching. Experimental Cell Research. 2001;268:104–14. 10.1006/excr.2001.5270 11461123

[10] Tondon A, Kaunas R. The direction of stretch-induced cell and stress fiber orientation depends on collagen matrix stress. Plos One. 2014;9:e89592 10.1371/journal.pone.0089592 24586898 PMC3933569

[11] Sears C, Kaunas R. The many ways adherent cells respond to applied stretch. Journal of Biomechanics. 2016;49:1347–54. 10.1016/j.jbiomech.2015.10.014 26515245

[12] Lee C-F, Haase C, Deguchi S, Kaunas R. Cyclic stretch-induced stress fiber dynamics—dependence on strain rate, rho-kinase and MLCK. BBRC. 2010;401:344–9. 10.1016/j.bbrc.2010.09.046 20849825

[13] Tondon A, Hsu H-J, Kaunas R. Dependence of cyclic stretch-induced stress fiber reorientation on stretch waveform. Journal of Biomechanics. 2012;45:728–35. 10.1016/j.jbiomech.2011.11.012 22206828

[14] Iba T, Sumpio BE. Morphological response of human endothelial cells subjected to cyclic strain in vitro. Microvascular Research. 1991;42:245–54. 1779881 10.1016/0026-2862(91)90059-k

[15] Wang JH-C, Goldschmidt-Clermont P, Yin FC-P. Contractility affects stress fiber remodeling and reorientation of endothelial cells subjected to cyclic mechanical stretching. Annals of Biomedical Engineering. 2000;28:1165–71. 11144977 10.1114/1.1317528

[16] Goldyn AM, Roioja BA, Spatz JA, Ballestrem C, Kemkemer R. Force-induced cell polarisation is linked to RhoA-driven microtubule-independent focal-adhesion sliding. Journal of Cell Science. 2009;122:3644–51. 10.1242/jcs.054866 19812308 PMC2758800

[17] Goldyn AM, Kaiser P, Spatz JA, Ballestrem C, Kemkemer R. The kinetics of force-induced cell reorganization depend on microtubules and actin. Cytoskeleton. 2010;67:241–50. 10.1002/cm.20439 20191565 PMC3638371

[18] Eltzner B, Wollnik C, Gottschlich C, Huckemann S, Rehfeldt F. The filament sensor for near real-time detection of cytoskeletal fiber structure. Plos One. 2015;10:e0126346 10.1371/journal.pone.0126346 25996921 PMC4440737

[19] Lichtenstein N, Geiger B, Kam Z. Quantitative analysis of cytoskeletal organization by digital fluorescent microscopy. Cytometry Part A. 2003;54A:8–18. 10.1002/cyto.a.10053 12820116

[20] Shah SA, Santago P, Rubin BK. Quantification of biopolymer filament structure. Ultramicroscopy. 2005;104:244–54. 10.1016/j.ultramic.2005.04.007 15961231

[21] Basu R, Liu C, Rohde GK. Localizing and extracting filament distributions from microscopy images. Journal of Microscopy. 2015;258:13–23. 10.1111/jmi.12209 25556529 PMC5890959

[22] Vindin H, Bischof L, Gunning P, Stehn J. Validation of an algorithm to quantify changes in actin cytoskeletal organization. Journal of Biomolecular Streening. 2014;19:345–68. 10.1177/1087057113503494 24019255

[23] Inoue S, Frank V, Hörning M, Kaufmann S, Yoshikawa HY, Madsen JP, et al Live cell tracking of symmetry break in actin cytoskeleton triggered by abrupt changes in micromechanical environments. Biomaterials Science. 2015;3:1539–11544. 10.1039/c5bm00205b 26347909

[24] Rehfeldt F, Brown AEX, Raab M, Cai S, Zajac AL, Zemel A, et al Hyaluronic acid matrices show matrix stiffness in 2D and 3D dictates cytoskeletal order and myosin-II phosphorylation within stem cells. Integrative Biology. 2012;4:422–30. 10.1039/c2ib00150k 22344328

[25] Zemel A, Rehfeldt F, Brown AEX, Discher DE, Safran SA. Optimal matrix rigidity for stress-fibre polarization in stem cells. Nature Physics. 2010;6:468–73. 10.1038/nphys1613 20563235 PMC2885792

[26] Drew NK, Eagleson MA, Baldo DB Jr, Parker KK, Grosberg A. Metrics for assessing cytoskeletal orientational correlations and consistency. Plos One. 2015;11:e1004190. 10.1371/journal.pcbi.1004190 PMC4388480 25849553

[27] Yoshigi M, Clark EB, Yost HJ. Quantification of stretch-induced cytoskeletal remodeling in vascular endothelial cells by image processing. Cytometry Part A. 2003;55A:109–18. 10.1002/cyto.a.10076 14505316

[28] Petroll WM, Cavanagh HD, Barry P, Andrews P, Jester JV. Quantitative analysis of stress fiber orientation during corneal wound contraction. Journal of Cell Science. 1993;104:353–63. 8505365 10.1242/jcs.104.2.353

[29] Lockett S, Verma C, Brafman A, Gudla P, Nandy K, Mimaky Y, et al Quantitative analysis of F-actin redistribution in astrocytoma cells treated with candidate pharmaceuticals. Cytometry Part A. 2014;85A:512–21. 10.1002/cyto.a.22442 PMC4385705 24515854

[30] Weichsel J, Herold N, Lehmann MJ, Kräusslich H-G, Schwarz US. A quantitative measure for alterations in the actin cytoskeleton investigated with automated high-throughput microscopy. Cytometry Part A. 2010;77A:52–63. 10.1002/cyto.a.20818 19899129

[31] Carlson MA, Thompson JS. Wound matrix attachment regulates actin content and organization in cells of the granulation tissue. Wound repair and regeneration. 2005;13:84–92. 10.1111/j.1067-1927.2005.130111.x 15659040

[32] Fuseler JW, Millette CF, Davis JM, Carver W. Fractal and image analysis of morphological changes in the actin cytoskeleton of neonatal cardiac fibroblasts in response to mechanical stretch. Microscopy and Analysis. 2007;13:133–43. 10.1017/S1431927607070225 17367553

[33] Greiner AM, Chen H, Spatz JA, Kemkemer R. Cyclic tensile strain controls cell shape and directs actin stress fiber formation and focal adhesion alignment in spreading cells. Plos One. 2013;8:e77328 10.1371/journal.pone.0077328 24204809 PMC3810461

[34] Cummins HZ, Pike ER. Photon Correlation and Light Beating Spectrocopy. New York: Plenum Press; 1974.

[35] Martins PC. Measurements and correlation functions. New York: Gordon and Breach; 1968.

[36] Zielinski A, Linnartz C, Pleschka C, Dreissen G, Springer R, Merkel R, et al Reorientation dyanmics and structural interdependencies of actin, microtubules and intermediate filaments upon cyclic stretch application. Cytoskeleton. 2018;75:385–94. 10.1002/cm.21470 30176121

[37] Mehta CR, Patel NR. SPSS Exact Tests 7.0 for Windows. Chicago, IL: SPSS Inc.; 1995.

[38] Hedges LV. Distribution Theory for Glass’s estimator of Effect Size and Related Estimators Journal of Educational Statistics. 1981;6(2):107–28.

[39] Zack GW, Rogers WE, Latt SA. Automatic measurement of sister chromatid exchange frequency. Journal of Histochemistry and Cytochemistry. 1977;25:741–53. 10.1177/25.7.70454 70454

[40] Niblack W. An introduction to digital image processing. Englewood Cliffs: Prentice-Hall International; 1986.

[41] Frykman P, Rogon TA. Anisotropy in pore networks analyzed with 2-D autocorrelation (variomaps). Computers and Geosciences. 1993;19:887–930.

[42] Press WH, Teukolsky SA, Vetterling WT, Flannery BP. Numerical Recipes in C The Art of Scientific Computing. Cambridge, UK: Cambridge University Press; 1992.

[43] Flyvbjerg H, Petersen HG. Error estimates on averages of correlated data. Journal of Chemical Physics. 1989;91:461–6.

[44] Wohland T, Rigler R, Vogel H. The Standard Deviation in Fluorescence Correlation Spectroscopy. Biophysical Journal. 2001;80:2987–99. 10.1016/S0006-3495(01)76264-9 11371471 PMC1301482

[45] Trepat X, Deng L, An SS, Navajas D, Tschumperlin DJ, Gerthoffer WT, et al Universal physical responses to stretch in the living cell. Nature. 2007;447:592–5. 10.1038/nature05824 17538621 PMC2440511

[46] Ramaswamy K, Park CY, Lin Y-C, Mead J, Jaspers RT, Trepat X, et al Reinforcement versus fluidization in cytoskeletal mechanoresponsiveness. Plos One. 2009;4(5):e5486 10.1371/journal.pone.0005486 19424501 PMC2675060

[47] Geiger B, Bershadsky AD, Pankov R, Yamada KM. Transmembrane extracellular matrix-cytoskeleton crosstalk. Nature Reviews in Molecular Cell Biology. 2001;2:793–805. 10.1038/35099066 11715046

[48] Kanchanawong P, Shtengel G, Pasapera AM, Ramko EB, Davidson MW, Hess HF, et al Nanoscale architecture of integrin-based cell adhesions. Nature. 2010;468:580–4. 10.1038/nature09621 21107430 PMC3046339

[49] Zaidel-Bar R, Ballestrem C, Kam Z, Geiger B. Early molecular events in the assembly of matrix adhesions at the leading edge of migrating cells. Journal of Cell Science. 2003;116:4605–13. 10.1242/jcs.00792 14576354

[50] Burridge K, Wittchen ES. The tension mounts: stress fibers as force-generating mechanotransducers. Journal of Cell Biology. 2013;200:9–19. 10.1083/jcb.201210090 23295347 PMC3542796

[51] Livne A, Geiger B. The inner workings of stress fibers—from contractile machinery to focal adhesions and back. Journal of Cell Science. 2016;129:1293–304. 10.1242/jcs.180927 27037413

[52] Tojkander S, Gateva G, Scheyzov G, Hotulainen P, Naumanen P, Martin C, et al A molecular pathway for myosin II recruitment to stress fibers. Current Biology. 2011;21:539–50. 10.1016/j.cub.2011.03.007 21458264

[53] Bershadsky AD, Chausovksy A, Becker E, Lyubimova A, Geiger B. Involvement of microtubules in the control of adhesion-dependent signal transduction. Current Biology. 1996;6:1279–89. 8939572 10.1016/s0960-9822(02)70714-8

[54] Krylyshkina O, Anderson KI, Kaverina I, Upmann I, Manstein DJ, Small JV, et al Nanometer targeting of microtubules to focal adhesions. Journal of Cell Biology. 2003;161:853–9. 10.1083/jcb.200301102 12782685 PMC2172972

[55] Small JV, Kaverina I. Microtubules meet substrate adhesions to arrange cell polarity. Current Biology. 2003;15:40–7. 10.1016/s0955-0674(02)00008-x 12517702

[56] Kaverina I, Rottner K, Small JV. Targeting, capture, and stabilization of microtubules at early focal adhesions. Journal of Cell Biology. 1998;142:181–90. 9660872 10.1083/jcb.142.1.181 PMC2133026

[57] Gonzales M, Weksler B, Tusruta D, Goldman RD, Yoon KJ, Hopkinson SB, et al Structure and function of a vimentin-associated matrix adhesion in endothelial cells. Molecular Biology of the Cell. 2001;12:85–100. 10.1091/mbc.12.1.85 11160825 PMC30570

[58] Tsuruta D, Jones JCR. The vimentin cytoskeleton regulates focal contact size and adhesion of endothelial cells subjected to shear stress. Journal of Cell Science. 2003;116:4977–84. 10.1242/jcs.00823 14625391

[59] Bershadsky AD, Tint IS, Svitkina TM. Association of intermediate filaments with vinculin-containg adhesion plaques of fibroblasts. Cell Motility and the Cytoskeleton. 1987;8:274–83. 10.1002/cm.970080308 3121191

[60] Salmon W, Adams MC, Waterman-Storer CM. Dual wavelength fluorescent speckle microscopy reveals coupling of microtubule and actin movements in migrating cells. Journal of Cell Biology. 2002;158:31–7. 10.1083/jcb.200203022 12105180 PMC2173033

[61] Coles CH, Bradke F. Coordinating neuronal actin-microtubule dynamics. Current Biology. 2015;25:R677–R91. 10.1016/j.cub.2015.06.020 26241148

[62] Rosales-Nieves AE, Johndrow JE, Keller LC, Magie CR, Pinto-Santini DM, Parkhurst SM. Coordination of microtubule and microfilament dynamics by Drosophila Rho1, Spire and Cappuccino. Nature Cell Biology. 2006;8:367–76. 10.1038/ncb1385 16518391 PMC1997291

[63] Basu R, Chang F. Shaping the actin cytoskeleton using microtubule tips. Current Opinion in Cell Biology. 2007;19:88–94. 10.1016/j.ceb.2006.12.012 17194581

[64] Leung CL, Sun D, Zheng M, Knowles DR, Liem RKH. Microtubule actin cross-linking factor (MACF): A hybrid of dystonin and dystrophin that can interact with the actin and microtubule cytoskeletons. Journal of Cell Biology. 1999;147:1275–85. 10601340 10.1083/jcb.147.6.1275 PMC2168091

[65] Wu X, Kodama A, Fuchs E. ACF7 regulates cytoskeletal-focal adhesion dynamics and migration and has ATPase activity. Cell. 2008;135:137–48. 10.1016/j.cell.2008.07.045 18854161 PMC2703712

[66] Stround MJ, Nazgiewicz A, McKenzie EA, Wang RA, Kammerer RA, Ballestrem C. GAS2-like proteins mediate communication between microtubules and actin through interactions with end-binding proteins. Journal of Cell Science. 2014;127:2672–82. 10.1242/jcs.140558 24706950 PMC4058111

[67] Chang L, Goldman RD. Intermediate filaments mediate cytoskeletal crosstalk. Nature Reviews in Molecular Cell Biology. 2004;5:601–13. 10.1038/nrm1438 15366704

[68] Gurland G, Gundersen GG. Stable, detyrosylated microtubules function to localize vimentin intermediate filaments in fibroblasts. Journal of Cell Biology. 1995;131:1275–90. 8522589 10.1083/jcb.131.5.1275 PMC2120637

[69] Svitkina TM, Verkhovsky AB, Borisy GG. Plectin sidearms mediate interaction of intermediate filaments with microtubules and other components of the cytoskeleton. Journal of Cell Biology. 1996;135:991–1007. 8922382 10.1083/jcb.135.4.991 PMC2133373

[70] Gyoeva FK, Gelfand VI. Coalignment of vimentin intermediate filaments with microtubules depends on kinesin. Nature. 1991;353:445–8. 10.1038/353445a0 1832745

[71] Prahlad V, Yoon M, Moir RD, Vale RD, Goldman RD. Rapid movements of vimentin on microtubule tracks: Kinesin-dependent assembly of intermediate filament networks. Journal of Cell Biology. 1998;143:159–70. 9763428 10.1083/jcb.143.1.159 PMC2132817

[72] Helfand BT, Chang L, Goldman RD. Intermediate filaments are dynamic and motile elements of cellular architecture. Journal of Cell Science. 2004;117:133–41. 10.1242/jcs.00936 14676269

[73] Helfand BT, Mikami A, Vallee RB, Goldman RD. A requirement for cytoplasmic dynein and dynactin in intermediate filament network assembly and organization. Journal of Cell Biology. 2002;157:795–806. 10.1083/jcb.200202027 12034772 PMC2173407

[74] Hookway C, Ding L, Davidson MW, Rappaport JZ, Danuser G, Gelfand VI. Microtubule-dependent transport and dynamics of vimentin intermediate filaments. Molecular Biology of the Cell. 2015;26:1675–86. 10.1091/mbc.E14-09-1398 25717187 PMC4436779

[75] Robert A, Herrmann H, Davidson MW, Gelfand VI. Microtubule-dependent transport of vimentin filament precursors is regulated by actin and by the concerted action of rho- and p21-activated kinases. FASEB Journal. 2016;28:2879–90. 10.1096/fj.14-250019 PMC4062827 24652946

