# Supplementary figures and images for: Unbiased pattern analysis reveals highly diverse responses of cytoskeletal systems to cyclic straining
Source: PLoS One. 2019 Mar 13;14(3):e0210570. doi: 10.1371/journal.pone.0210570 (PMC6415792; doi:10.1371/journal.pone.0210570)

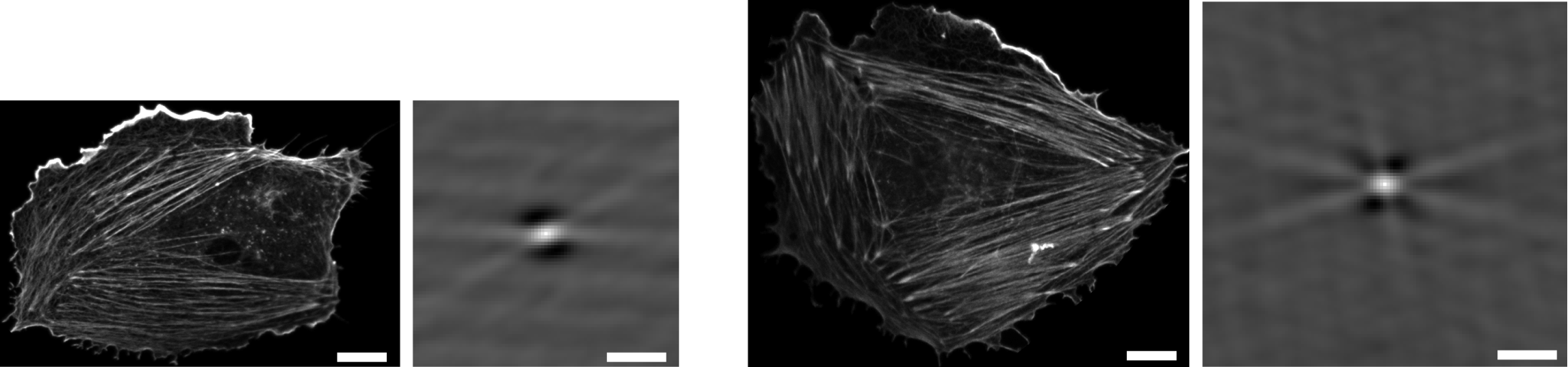

Supplement: S1 Fig — Shown are actin cytoskeletons and correlograms of control cells (no straining) exhibiting bimodal (left pair) or trimodal (right pair) order of filaments. Scale bars, 10 μm for micrographs, 2 μm for correlograms. Logarithmic lookup table for correlograms. (TIF) [file pone.0210570.s001.tif]

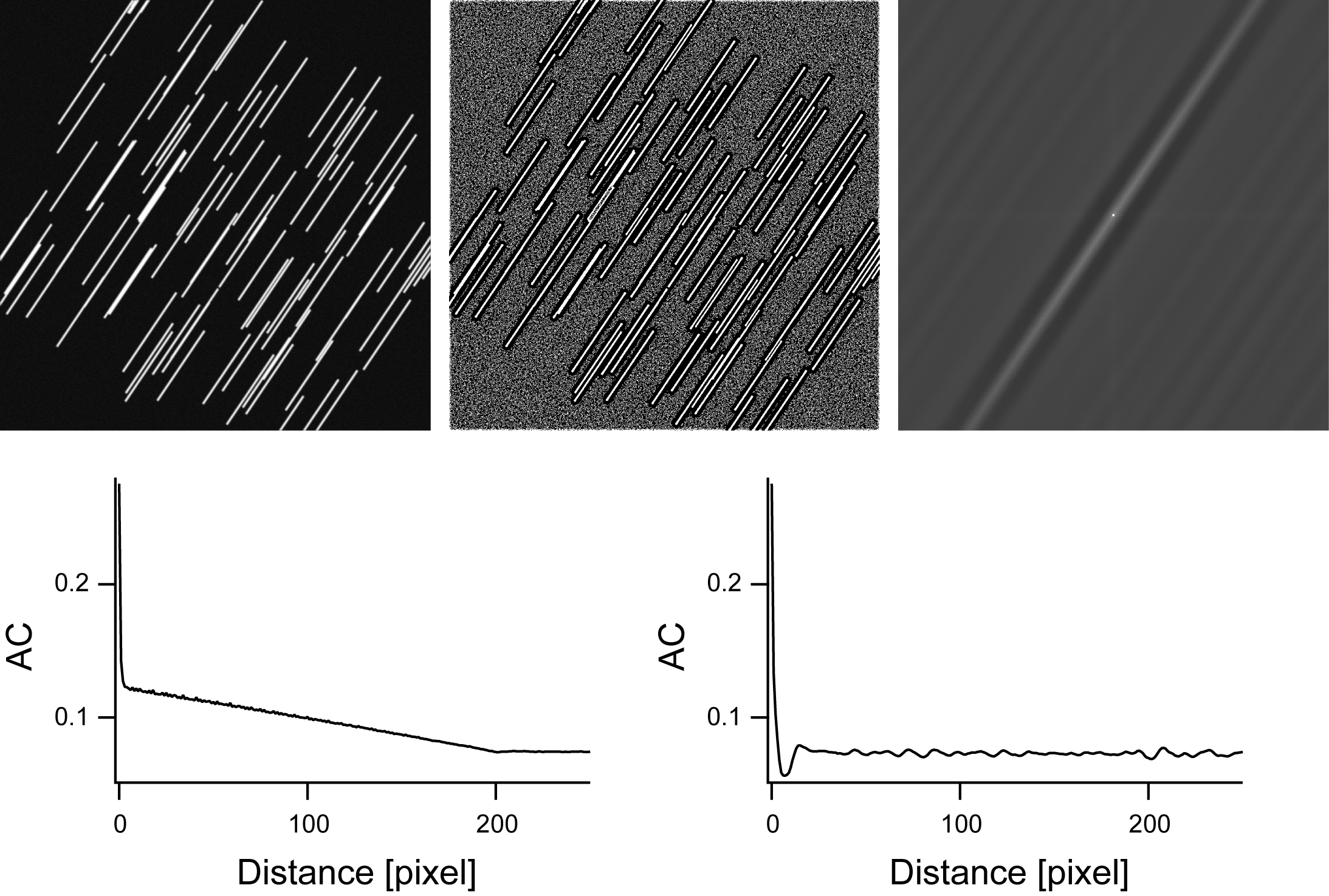

Supplement: S2 Fig — Hundred rectangles (5 by 200 pixel) oriented under 34° to the vertical axis with realistic intensity noise added (top left) were segmented (top middle) and autocorrelated (top right, only central 201 x 201 pixels are shown). Line profiles of the correlogram along (bottom left) and normal (bottom right) to the feature orientation are also shown. Note the dark halos around lines and spot-noise in the segmentation. These were a filter-induced artifact and caused a pronounced depression of the correlogram in direction perpendicular to the lines. Fiber length was encoded in the slope of the linear decay of the correlogram along fiber direction. (TIF) [file pone.0210570.s002.tif]
